# Detection of l-arginine

**DOI:** 10.1039/d6ra06294f

**Published:** 2026-11-05

**Authors:** Wafaa Waleed Al-Qaysi, Rawaa A. Faris, Alyaa Khider Abbas

**Affiliations:** a Department of Chemistry, College of Science, University of Baghdad Baghdad Iraq wafa.w@sc.uobaghdad.edu.iq rawaa@ilps.uobaghdad.edu.iq alyaa.abbas@sc.uobaghdad.edu.iq; b Institute of Laser for Postgraduate Studies, University of Baghdad Baghdad Iraq

## Abstract

A microfluidic colorimetric sensing platform was developed which is rapid, sensitive and environmentally friendly for the selective determination of l-arginine based on its supramolecular interaction with nanocurcumin. The sensing mechanism is based on the formation of a stable nanocurcumin–l-arginine assembly through hydrogen bonding, electrostatic attraction and cation–π interactions, which leads to the appearance of a characteristic absorption band at 550 nm and a visible color change from yellow to deep red. The analytical performance of the sensing system was systematically optimized by studying the critical parameters such as nanocurcumin concentration, flow rate, temperature and reaction conditions. The incorporation of nanocurcumin with microfluidic technology showed a remarkable improvement in mass-transfer efficiency, reagent usage, waste generation and analytical sensitivity, which is in line with the principles of green analytical chemistry. The microfluidic platform exhibited excellent linearity under optimized conditions with the regression equation *y* = 0.400*x* + 0.0050 and a correlation coefficient (*R*^2^) of 0.9993 in the concentration range of 0–20 µmol L^−1^. The developed microfluidic platform showed a limit of detection (LOD) and limit of quantification (LOQ) of 0.825 nmol L^−1^ and 2.5 nmol L^−1^, respectively, which were about two-fold lower than the conventional batch method. The proposed method showed high precision, good repeatability and excellent selectivity towards l-arginine in the presence of potential interfering species. The developed platform was successfully applied to the analysis of pharmaceutical formulations and biological samples, with satisfactory recovery values and low matrix effects. In addition, the greenness and whiteness assessments showed that the proposed approach was environmentally sustainable. The developed nanocurcumin based microfluidic platform is a simple, rapid, cost-effective, eco-friendly analytical approach for the routine determination of l-arginine and represents a promising platform for future biomedical and point-of-care sensing applications.

## Abstract 2

A nanocurcumin–l-arginine supramolecular assembly triggers a selective yellow-to-red colour change and a new absorbance band at 550 nm, enabling rapid, sensitive microfluidic colorimetric detection of l-arginine.

## Graphical abstract

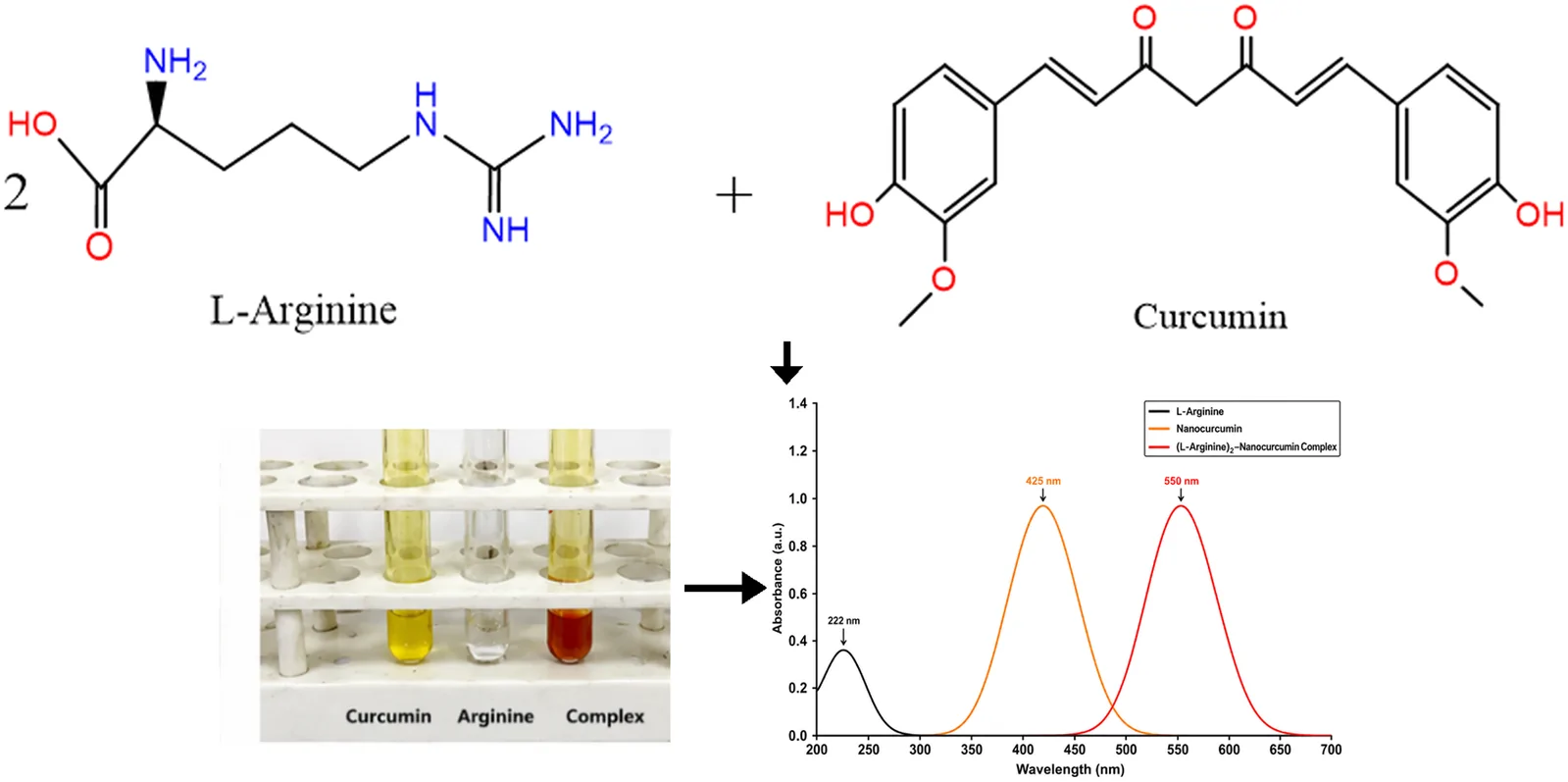

## 1. Introduction

l-Arginine is a semi-essential amino acid with a fundamental role in many physiological and metabolic pathways such as nitric oxide biosynthesis, immune regulation, protein metabolism, cellular signaling, and vascular homeostasis. Because of its important biomedical and pharmaceutical applications, the precise determination of l-arginine has received much attention in clinical diagnostics, pharmaceutical quality control, food analysis, and biochemical studies.^1^ There are several analytical methods available for l-arginine determination, such as high-performance liquid chromatography (HPLC),^2^ HPLC coupled with mass spectrometry (HPLC-MS/MS),^3^ liquid chromatography-tandem mass spectrometry (LC-MS/MS),^4^ capillary electrophoresis (CE),^5^ ion-exchange chromatography (IEC),^6^ fluorometric methods,^7^ and spectrophotometric assays.^8^ These methods are adequate in terms of sensitivity and selectivity, but many of them are complex and require expensive instrumentation, complex sample preparation, highly trained staff, and significant amounts of organic solvents and reagents. Thus, the need for the development of rapid, cost-effective, miniaturized, and environmentally friendly analytical platforms is still a research goal.^9^

However, colorimetric sensing has become a popular analytical approach in recent years because of its simplicity, quick response, inexpensive, and ability to be used for naked-eye detection without the use of a sophisticated instrument. These benefits render colorimetric sensors an ideal choice for use in point-of-care diagnostics, on-site environmental monitoring, pharmaceutical quality control, and food safety analysis. Moreover, the incorporation of nanomaterials into colorimetric sensing platforms has significantly enhanced the sensitivity, selectivity, and signal amplification of the analytical platform using their optical and physicochemical properties. Thus, the development of colorimetric sensors that can be based on nanomaterials has emerged as one of the most rapidly growing research fields in analytical chemistry, providing viable alternatives to traditional instrumental methods and contributing to the development of portable and environmentally-friendly sensing systems.^10–12^ The recent development of nanotechnology and microfluidics has opened new avenues for the development of highly efficient sensing systems.^13^ The optical sensors based on nanomaterials have been given much attention as analytical tools because of their high surface-to-volume ratio, unique optical properties, fast response time, and high interaction efficiency.

Curcumin is one of the most promising nanomaterials for sensing applications because of its long-conjugated structure, outstanding optical properties, and multiple reactive functional groups that can be involved in molecular recognition processes. The natural polyphenolic compound curcumin (1,7-bis-(4-hydroxy-3-methoxyphenyl)-hepta-1,6-diene-3,5-dione) extracted from *Curcuma longa* has been extensively used in optical and colorimetric sensing applications due to its chromophoric system and its versatile chemical reactivity.^14^ The conversion of curcumin into nanoscale form leads to better dispersibility, stability, surface activity and interaction efficiency, which leads to better sensing performance. Furthermore, recent studies have demonstrated that nanocurcumin can serve as an efficient sensing nanomaterial because of its favorable optical properties, chemical stability, and high analytical sensitivity, making it an attractive candidate for developing environmentally friendly optical sensing platforms.^15^

Microfluidic technology has several benefits over traditional analytical techniques, such as low reagent consumption, high mixing efficiency, short diffusion path, quick analysis, portability, and low waste generation. The combination of nanomaterials and microfluidic platforms is thus a promising approach for the next generation of analytical systems with high sensitivity and environmental friendliness. Although the use of nanomaterials in sensing platforms has been gaining interest, there are few reports on the direct colorimetric determination of l-arginine using nanocurcumin incorporated in a microfluidic analytical device.^16,17^ Current approaches often involve costly instrumentation, complex fabrication processes, or sophisticated recognition elements that can restrict the use of the approach in a routine manner. In our previous studies, we have successfully developed several environmentally friendly optical sensing systems based on nanomaterials such as nano azo compounds for pH sensing,^18^ Zn(ii)-based nano sensors,^19^ and Ni(ii)-based colorimetric optical fiber sensors.^20^ These studies laid the groundwork for the design of functional nanostructured sensing materials and motivated the design of the present eco-friendly nano-curcumin microfluidic platform for the selective determination of l-arginine.

To our knowledge, this is the first work that combines nanocurcumin with a microfluidic platform for UV-Vis spectroscopy for the rapid and selective colorimetric determination of l-arginine at 550 nm by supramolecular interaction. The proposed sensing strategy is based on the formation of a supramolecular nanocurcumin–l-arginine assembly, which gives a unique optical response at 550 nm. The continuous-flow microfluidic configuration reduces reagent consumption, reduces analysis time, increases mass-transfer efficiency, and allows for precise control of reaction conditions, leading to increased analytical sensitivity and reliability. The platform was optimized systematically by studying the effect of nanocurcumin concentration, flow rate and temperature and successfully validated for the determination of l-arginine in pharmaceutical formulations and biological samples (serum and urine). Furthermore, the analytical performance, selectivity, greenness and applicability of the proposed platform were thoroughly evaluated, which showed its potential as a simple, cost-effective, environmentally friendly and reliable analytical tool for routine l-arginine analysis.

## 2. Materials and reagents

### 2.1 Instruments

Fourier-transform infrared (FTIR) spectra were obtained with a Shimadzu FTIR-8400S spectrophotometer (Shimadzu Corporation, Japan). The free ligand was analyzed as KBr pellets over the wavenumber range of 400–4000 cm^−1^. The UV-Visible absorption spectra were recorded at room temperature with a Shimadzu UV-1800 spectrophotometer (Shimadzu Corporation, Japan) in the range of 190–1100 nm. The surface morphology and nanoscale roughness were analyzed by atomic force microscope (AFM; CoreAFM, Nanosurf AG, Switzerland). The surface morphology and particle-size characteristics were investigated by field-emission scanning electron microscopy (FE-SEM) using a TESCAN MIRA3 microscope, and the commercial CO_2_ laser system used to engrave the PMMA substrate is air-cooled (Liaocheng JK-4060 Laser Engraving and Cutting Machine, China). The laser operates in continuous wave mode at a frequency of 5 kHz, with a wavelength of 10.6 Im and a maximum output power of 60 watt. The beam intensity exhibits a near-Gaussian distribution. For the fabrication of the Y-shaped microfluidic chips, the laser power is 60 watt, and the beam travel speed can be set to 250, 350, or 500 mm s^−1^.

### 2.2 Chemicals and reagents

Chemicals and reagents used in this study were analytical-reagent grade and used as received without further purification. l-Arginine (98%) and curcumin (98%) were purchased from Fluka (Sigma-Aldrich, Switzerland) and Sigma-Aldrich (molecular weight = 174.20 g mol^−1^ and 368.38 g mol^−1^, respectively), respectively. Ammonium sulfate, (NH_4_)_2_SO_4_ (98% purity) was purchased from Fluka (Sigma-Aldrich, Switzerland; molecular weight = 132.14 g mol^−1^) and was used for protein precipitation in serum sample preparation. The inorganic salts used to prepare the interfering metal-ion solutions were silver nitrate (AgNO_3_), lead(ii) nitrate [Pb(NO_3_)_2_], bismuth(iii) nitrate pentahydrate [Bi(NO_3_)_3_·5H_2_O], cadmium nitrate tetrahydrate [Cd(NO_3_)_2_·4H_2_O], and mercury(ii) chloride (HgCl_2_), serving as the sources of Ag^+^, Pb^2+^, Bi^3+^, Cd^2+^, and Hg^2+^, respectively. All inorganic salts were purchased from Fluka (Sigma-Aldrich, Switzerland). Selectivity and interference studies were carried out using l-histidine, glycine, l-alanine, urea and uric acid, which were also purchased from Fluka (Sigma-Aldrich, Switzerland). Nanocurcumin was synthesized and characterized in our laboratory as reported earlier.^15^ All standard, reagent and sample solutions were prepared using distilled water.

### 2.3 Preparation of standard and reagent solutions of l-arginine

Standard and reagent solutions of l-arginine were prepared as follows: a stock standard solution of l-arginine (100 µmol L^−1^) was prepared by accurately weighing 0.178 mg of l-arginine (≥98%, Fluka/Sigma-Aldrich; molecular weight = 174.20 g mol^−1^), dissolving it in distilled water and making up to a final volume of 10 mL. Appropriate dilutions of the stock solution with distilled water were used to prepare working solutions.

### 2.4 Synthesis and characterization of nanocurcumin

Turmeric rhizome powder was first extracted with ethanol following our previously published procedure^15^ to obtain curcumin. The plant material was extracted twice with ethanol (99.9%) for 24 h with continuous shaking at 37 °C. The extracts were filtered and concentrated using a rotary evaporator at 40 °C and 50 mbar. The extracted curcumin was then used for the synthesis of nanocurcumin by the pH induced precipitation method as described in our previous publication.^15^ A 2.0 × 10^−3^ mol per L curcumin solution was prepared in acetone and 5 mL of this solution was added dropwise to 45 mL of deionized water with continuous stirring at 500 rpm. The pH was adjusted to about 10 with 1 mol per L NaOH and stirred for 60 min, then 1 mol per L HCl was added dropwise until pH 4 was reached and stirred for another 60 min. The pH was then adjusted to 7 with NaOH and the suspension was stirred for a further 60 min at 25 °C. The formed nanoparticles were centrifuged at 10 000 rpm for 15 min, washed twice with deionized water and dried at 40 °C for 24 h. The dried nanocurcumin was kept in a sealed container for further use.

The nanocurcumin prepared in the present study was characterized by UV-Visible spectroscopy, scanning electron microscopy (SEM, Fig. S1), atomic force microscopy (AFM, Fig. S2), and Fourier-transform infrared spectroscopy (FTIR). The characterization data obtained were used to assess the optical properties, morphology, particle size and characteristic functional groups of the synthesized nanocurcumin and compared with the previously reported data.^15^ A nanocurcumin stock solution with a concentration of 100 µmol L^−1^ was prepared by accurately weighing 0.376 mg of the dried nanocurcumin powder (C_21_H_20_O_6_, 98%, Sigma-Aldrich; molecular weight = 368.38 g mol^−1^) and dissolving it in ethanol absolute to a final volume of 10 mL. The prepared stock solution was used in the present study for preparing the required working solutions and the microfluidic colorimetric sensing platform.

### 2.5 Spectrophotometric procedure

The proposed method was based on the interaction between l-arginine and nanocurcumin under optimized conditions to form a stable dark red complex. In a 5 mL volumetric flask, 0.3 mL of nanocurcumin solution was mixed with 0.7 mL of l-arginine solution, followed by dilution to volume. The mixture was vortexed for 2 min, and the absorbance was measured after 1 min at 550 nm against a reagent blank.

### 2.6 Calibration procedure

Calibration curves were constructed using a series of l-arginine standard solutions within the concentration range of 0–20 µmol L^−1^ under optimized experimental conditions. Linear regression analysis was used to evaluate analytical parameters including sensitivity, linearity, and correlation coefficient.

For microfluidic analysis, the analyte and reagent streams were continuously merged within a single-channel microfluidic system under optimized flow condition.

### 2.7 Design and fabrication of microfluidic system

Two channel microfluidic system with two inlets and one outlet was designed and fabricated. Optimization of the microchannel design, infusion pressure and flow time was performed to achieve stable and reproducible operation. The microfluidic chip was made of polymethyl methacrylate (PMMA). The chip design was created using SolidWorks software, and the layout of the microfluidic chip is shown in Fig. 1A. The microfluidic chip had two inlet channels and one outlet channel. A CO_2_ laser cutting system was used to fabricate the PMMA chip. Two PMMA layers of thickness 2 mm and size 2 cm × 5 cm were prepared. The first layer had two inlet openings and one outlet opening, while the second layer had the microchannel pattern laser engraved, as illustrated in Fig. 1B. The two layers were then joined together to create the full microfluidic device.

**Fig. 1:**
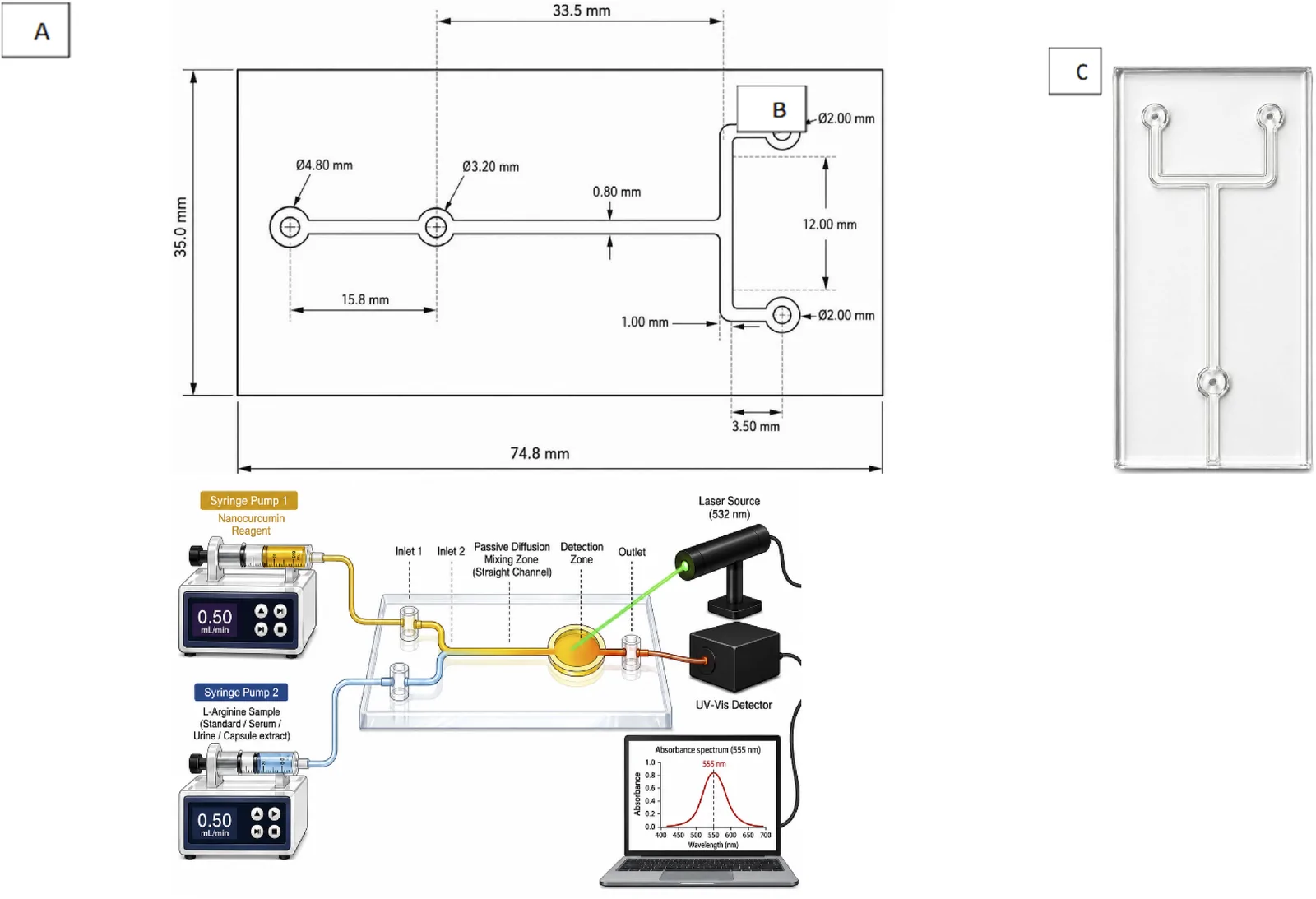
(A) SolidWorks design of the microfluidic chip with 2 mm thickness. (B) Final fabricated microfluidic chip. (C) Schematic representation of the nanocurcumin microfluidic sensing platform based colorimetric determination of l-arginine in pharmaceutical and biological samples.

### 2.8 Laser-microfluidic colorimetric detection system

Using nanocurcumin as a chromogenic sensing reagent, the developed microfluidic sensing platform was applied to the colorimetric determination of l-arginine. The experimental system comprised (1) a dual syringe pump, (2) a PMMA-based microfluidic chip, and (3) an optical density (OD) detection system at 550 nm. The first syringe contained the nanocurcumin reagent, and the second syringe contained the l-arginine standard solution or the biological sample and was injected into the microfluidic channel. In the microchannel, a stable nanocurcumin–arginine complex was formed, resulting in a red color with a maximum absorption at 550 nm (Fig. 1C). The developed microfluidic platform was applied to qualitative and quantitative analysis for determination of l-arginine in pharmaceutical capsule formulations and biological samples like human serum and urine.

The absorbance response was then continuously monitored under optimized flow conditions for the rapid and reproducible determination of l-arginine concentration. Analytical performance was assessed by linearity, limit of detection (LOD), limit of quantification (LOQ), repeatability and recovery. The results were compared with those of a conventional UV-Vis spectrophotometric method in terms of reagent consumption, analysis time, and analytical sensitivity.

### 2.9 Sample preparation and analysis of real samples

Experiments involving human serum and urine samples were reviewed and approved by the Institutional Research Ethics Committee of University of Baghdad (approval no. CSFC/0626/0058) on 10 June 2026. All procedures were performed in accordance with the Declaration of Helsinki. Informed consent was obtained from all participants prior to sample collection.

Commercial l-arginine capsules (500 mg declared active content) were purchased from three different suppliers: (1) Puritan's Pride l-Arginine (USA); (2) Mega Strength l-Arginine (USA); and (3) NOW Foods l-Arginine (USA). The average capsule content mass was determined for each brand by accurately weighing 12 capsules and emptying them. A sample of the powder was accurately weighed and transferred to a beaker, and dissolved in distilled water to the equivalent of 0.1742 g of active l-arginine. Insoluble excipients were removed by filtering the mixture through Whatman No. 42 filter paper. The filtrate was transferred quantitatively to a 10 mL volumetric flask and diluted to the mark with distilled water to give the primary sample stock solution of 17.420 µg mL^−1^ (100 µM). The samples were then diluted to fit into the linear working range of the instrument (0–8 µmol L^−1^) before spectrophotometric measurements were taken. For biological matrix analysis, human serum samples were first deproteinized by mixing equal volumes of serum and ammonium sulfate (NH_4_)_2_SO_4_ (1 : 1 v/v) followed by centrifugation at 10 000 rpm for 10 min to precipitate proteins. The clear supernatant was collected and diluted 10-fold (1 : 10 dilution factor) with phosphate-buffered saline (PBS, pH 7.4) to bring the analyte concentration into the linear dynamic range (0–4 µmol L^−1^).^21^ The fresh human urine samples were filtered through a 0.22 µm membrane filter to remove particulate debris, and then diluted 20-fold (1 : 20 dilution factor) with deionized water to reduce the high salinity background effects before analysis in the linear range of the instrument (0–6 µmol L^−1^).

Each experiment and analytical measurement was repeated three times (*n* = 3) and the results are presented as mean ± standard deviation (SD).

## 3. Result and discussion

### 3.1 FTIR spectrum of nanocurcumin, and nanocurcumin–l-arginine complex

FTIR spectroscopy was employed to investigate the molecular interactions occurring between nanocurcumin and l-arginine are shown in Fig. 2. The spectrum of pure l-arginine (Fig. 2A) shows a wide absorption band of the *ν*(O–H) and *ν*(N–H) stretching vibration in the region between 3330 cm^−1^ and 3060 cm^−1^. The *ν*(C–H) stretching mode is observed at 2947 cm^−1^, and asymmetric carboxylate *ν*(COO^−^) stretching mode is seen as a prominent band at 1655 cm^−1^, and 1410 cm^−1^, respectively, confirming the zwitterionic structure of l-arginine, and the *ν*(N–H) bending mode is observed as a prominent band at 1577 cm^−1^. Other characteristic bands are assigned to the *ν*(C–N), *ν*(C–O) and *ν*(N–H) vibrational modes between 1450–1220 cm^−1^, while the lower wavenumber absorptions are assigned to fingerprint skeletal vibrations of the amino acid. The bands between 1120 and 1040 cm^−1^ were attributed to C–N stretching vibrations, which are characteristic of the amino acid structure.^22^ In Fig. 2B, the spectrum of nanocurcumin showed the characteristic bands of O–H stretching (3508 cm^−1^), aliphatic C–H stretching (2921 and 2850 cm^−1^) and C=O stretching of the β-diketone carbonyl stretching near 1625 cm^−1^. The other bands in the range 1510–1026 cm^−1^ were assigned to aromatic C=C and C–O stretching vibrations, suggesting that the structure of curcumin did not change after nano formulation. These spectral features are in agreement with the molecular structure of curcumin and indicate that the functional framework of curcumin is preserved after nano formulation.

**Fig. 2:**
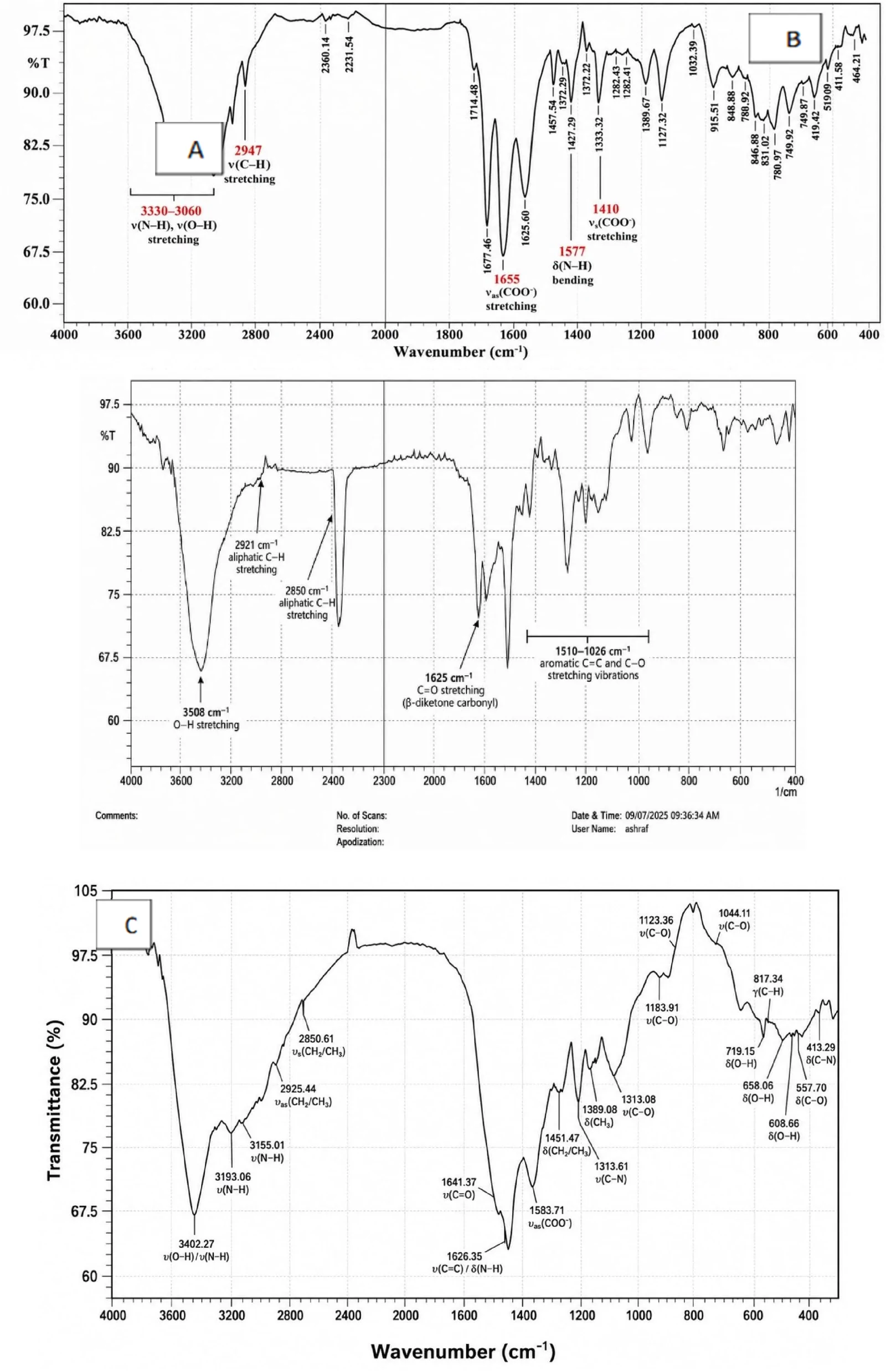
FTIR spectra of (A) pure l-arginine, (B) nanocurcumin, & (C) nanocurcumin–l-arginine complex.

In Fig. 2C, the FTIR spectrum of nanocurcumin–l-arginine complex showed significant broadening of the bands and slight shifts in some characteristic absorption bands as compared to the individual components Fig. 2. The most notable change was in the carbonyl stretching vibration which changed from 1625 to 1641 cm^−1^. Also, significant broadening was observed in the O–H/N–H stretching region, which suggests that hydrogen-bonding interactions are involved. The intensity of the carboxylate-related bands also varies, further supporting intermolecular interactions between nanocurcumin and l-arginine. The spectral changes indicate that a supramolecular complex is formed, which is stabilized by hydrogen bonding and electrostatic interactions. Importantly, no new absorption bands due to covalent bond formation were observed, suggesting that the interaction was non-covalent. These supramolecular interactions are reversible and desirable for sensing applications because they allow for the rapid formation of the complex without compromising the structure of the sensing material.^23^

Overall, these FTIR spectral changes validate the chemical modulation of functional groups on the complexation of nanocurcumin with l-arginine.^24,25^ The results are in good agreement with the proposed supramolecular recognition mechanism and with the 1 : 2 (nanocurcumin : l-arginine) stoichiometry obtained by Job's method of continuous variation as well as with the observed bathochromic shift in the UV-Vis spectrum.

### 3.2 UV-vis spectroscopy investigation

UV-Vis spectroscopy provided direct evidence for the interaction between nanocurcumin and l-arginine are shown in Fig. 3. Pure l-arginine has a characteristic absorption band in the deep-UV region centered at ∼222 nm, as seen in (Fig. 3A and S3). The absorption of l-arginine in the visible spectral range (400–800 nm) is negligible as expected from the colorless aqueous solution of l-arginine, which is due to the absence of π → π* electronic transitions in its functional groups and guanidinium moiety,^26^ (Fig. 3B and S4) nanocurcumin exhibited a characteristic absorption maximum at approximately 425 nm, attributed to π → π* transitions within its extended conjugated structure.^27^ In (Fig. 3C), following interaction with l-arginine, a new absorption band appeared at 550 nm accompanied by a substantial increase in absorbance intensity, the observed bathochromic shift indicates modification of the electronic environment of nanocurcumin as a consequence of supramolecular complex formation. Similar spectral shifts have been reported for curcumin-based sensing systems involving charge-transfer interactions and molecular aggregation processes. The intensity of the 550 nm band increased progressively with increasing l-arginine concentration, demonstrating a concentration-dependent optical response suitable for quantitative analysis. The excellent linearity observed confirms that complex formation proceeds in a reproducible manner across the investigated concentration range.

**Fig. 3:**
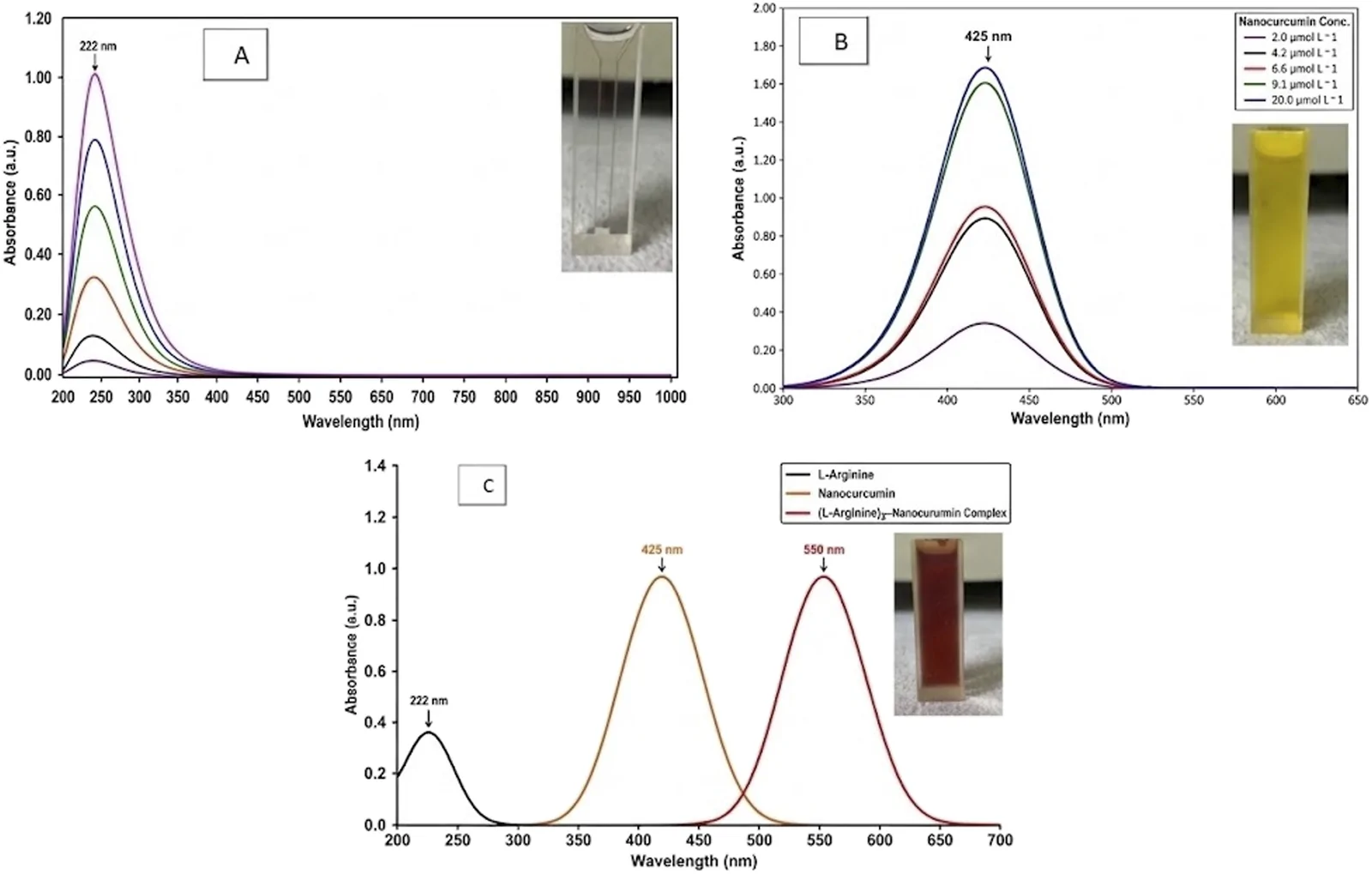
UV-Vis absorption spectra recorded under optimized experimental conditions: (A) pure l-arginine solution (10 µmol L^−1^), displaying a characteristic maximum absorption at *λ*_max_ ≈ 222 nm with absorbance ∼0.365 a.u.; (B) nanocurcumin probe solution (10 µmol L^−1^, *λ*_max_ = 425 nm); and (C) l-arginine–nanocurcumin complex solution formed at a 1 : 2 stoichiometry (10 µmol per L nanocurcumin + 20 µmol per L l-arginine) determined *via* Job's method of continuous variation, displaying a pronounced bathochromic red shift to *λ*_max_ = 550 nm. All spectra were acquired at room temperature (25 °C) using a Shimadzu UV-1800 spectrophotometer.

Under optimized conditions, pure l-arginine (10 µmol L^−1^) exhibited a maximum absorbance of ∼0.365 a.u. at 222 nm, while the nanocurcumin probe (10 µmol L^−1^) displayed its characteristic absorption maximum at 425 nm. Upon interaction with l-arginine (20 µmol L^−1^), the formation of the 1 : 2 nanocurcumin–l-arginine complex resulted in a prominent bathochromic shift of the absorption maximum from 425 nm to 550 nm (absorbance ∼1.12 a.u), visually confirming complexation.

### 3.3 Proposed colorimetric sensing mechanism of l-arginine

The interaction between nanocurcumin and l-arginine is suggested to be a stable supramolecular assembly, which is mainly based on hydrogen bonding and electrostatic attraction. l-Arginine has a positive guanidinium group that shows high affinity with the electron-rich oxygen-containing functionalities of nanocurcumin such as phenolic hydroxyl, enolic hydroxyl, and β-diketone carbonyl groups. The amino/guanidinium groups of l-arginine form multiple N–H⋯O hydrogen bonds with the oxygen donor groups of nanocurcumin and electrostatic attraction also contributes to the stabilization of the molecular assembly. These interactions cause the electronic environment of the extended π-conjugated curcumin framework to be disturbed, which leads to increased electron delocalization and redistribution of electron density. As a result, a new absorption band appears at 550 nm and a clear colour change from yellow to deep red is observed. The optical response obtained is the foundation of the proposed nanocurcumin colorimetric sensing mechanism for the determination of l-arginine as seen in Scheme 1.^24^

**Scheme 1:**
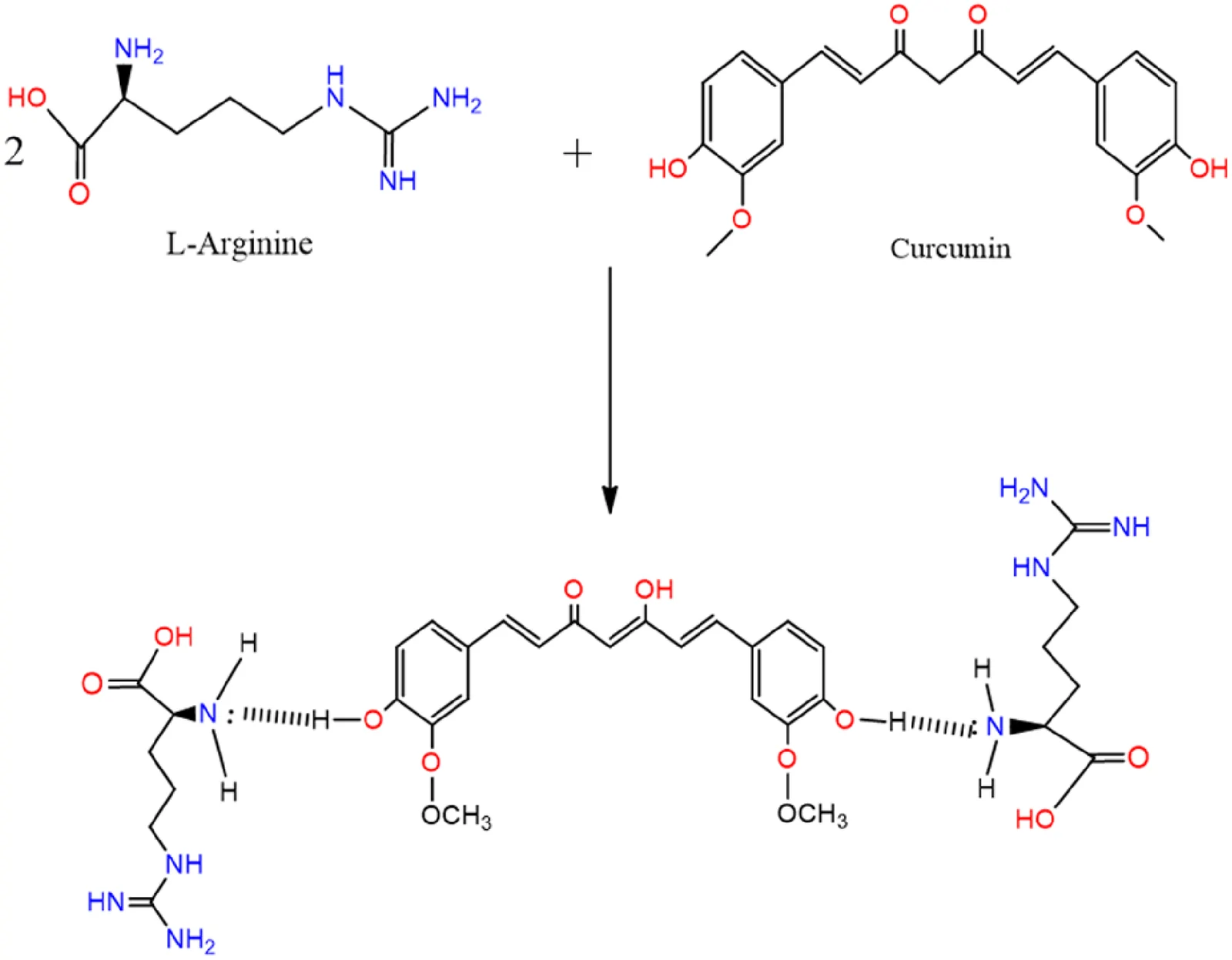
Proposed supramolecular interaction mechanism between nanocurcumin and l-arginine.

### 3.4 Investigation of nanocurcumin–arginine compound stability

#### 3.4.1 Effect of nanocurcumin concentration

The effect of nanocurcumin concentration on complex formation with a fixed concentration of l-arginine (20 µmol L^−1^) was investigated at *λ* = 550 nm (Fig. S5). The absorbance was found to be linearly proportional to the concentration of nanocurcumin up to 10 µmol L^−1^ (*A* = 1.39), suggesting progressive complexation. The absorbance plateaued beyond 10 µmol L^−1^, indicating that the binding sites were saturated. Thus, 10 µmol L^−1 ^was chosen as the optimum nanocurcumin concentration. The bathochromic shift to 550 nm is attributed to hydrogen bonding and electrostatic interactions between the phenolic hydroxyl groups of nanocurcumin and the amino/guanidinium groups of l-arginine, which enable charge-transfer interactions.^28^

#### 3.4.2 Effect of temperature

The stability and complexation of nanocurcumin–arginine complex was investigated at *λ* = 550 nm under the influence of temperature (15–50 °C) (Fig. S6). The absorbance was found to be constant between 15 and 25 °C (*A* ≈ 0.97), and then rapidly rose to a maximum value of 1.79 at 32 °C, which corresponds to optimal complex formation and energy-driven binding kinetics. At temperatures above 32 °C, a significant decrease in absorbance was seen, with a value of 0.52 at 50 °C. This reduction is due to the thermal dissociation of the complex and the loss of weak non-covalent interactions (*e.g.*, hydrogen bonding and electrostatic interactions) that result in less charge transfer efficiency. Therefore, the optimum reaction temperature was chosen as 32 °C.^29^

#### 3.4.3 Effect of time, & UV-irradiation on compound stability

The stability of compounds in the presence of time and UV-irradiation is discussed. The kinetic stability and photostability of the nanocurcumin–arginine complex were investigated for 200 min under ambient light and continuous UV irradiation (*λ* = 550 nm, Fig. S7). The absorbance under ambient conditions (time curve) showed a gradual decrease from 1.89 to 1.43 over 200 min and then levelled off after 100 min. A slight decrease in absorbance was seen from 1.78 to 1.23 under UV exposure (irradiation curve), which suggests minor photodegradation of the curcumin chromophore. The overall long-term stability of the complex was good, and the photostability was reasonable, with most of the optical response remaining within the 200 min monitoring window.

#### 3.4.4 Evaluation of linearity, precision, and detection capability

The analytical performance of the nanocurcumin-based optical probe for the determination of l-arginine was evaluated under optimized conditions at *λ* = 550 nm (Fig. S8 and Table S1). The calibration plot was linear over the concentration range of 0–10 µmol L^−1^ with a high correlation coefficient (*R*^2^ = 0.9975) and regression equation *A* = 0.1026*C* + 0.0327. The method exhibited exceptional sensitivity with a limit of detection (LOD) of 2.92 nmol L^−1^ (3.3*σ*/*m*) and a limit of quantification (LOQ) of 9.75 nmol L^−1^ (10*σ*/*m*). The low relative standard deviation (RSD = 0.2%, *n* = 3 at 6 µmol L^−1^), indicated high reproducibility and reliability for micro-molar and nano-molar l-arginine sensing.

#### 3.4.5 Analytical application in real samples

The developed spectrophotometric method was evaluated for the quantitative determination of l-arginine in pharmaceutical capsules, human serum and urine samples (Fig. S9). The linear responses were obtained for all sample matrices with good correlation coefficients (*R*^2^ = 0.9985 for capsules, 0.9918 for serum, and 0.9989 for urine) over the spiked concentration range (1–5 µmol L^−1^). Good analytical precision was achieved with acceptable RSD values (RSD < 4%). Minor matrix interferences are responsible for slight absorbance changes at lower concentrations (1–2 µmol L^−1^) in biological fluids, especially urine.

Overall, the high recoveries and precision confirm the reliability and suitability of the proposed nanocurcumin probe for routine clinical and pharmaceutical analysis.

## 4. Microfluidic chips

### 4.1 Optimization of microfluidic sensing parameters (flow rate and temperature)

The analytical performance of the nanocurcumin microfluidic sensing platform was assessed by optimizing the critical operational parameters that influence the formation and stability of the nanocurcumin–arginine complex at 550 nm. The absorbance response and sensing stability were systematically studied as a function of flow rate and temperature in a continuous-flow microfluidic environment. The absorbance and relative response of the nanocurcumin–arginine complex in the microfluidic system is plotted against flow rate in Fig. 4A. The absorbance gradually increased as the flow rate increased, and reached a maximum value at 50 µL min^−1^, which means that the interaction and mixing efficiency is optimal inside the microchannel. The absorbance was found to be lower at higher flow rates because of lower residence time and incomplete complex formation. The relative response showed a similar trend, further demonstrating the importance of flow conditions in the control of the efficiency of microfluidic sensing. The absorbance stability of the nanocurcumin–arginine complex at 550 nm was evaluated at various temperatures and is presented in Fig. 4B. The absorbance slowly decreased as the temperature increased, suggesting partial destabilization of the sensing complex at high temperatures. The relative stability showed a similar trend, which further demonstrates the significance of temperature control to ensure the stability of the sensing and analytical performance in the microfluidic platform.

**Fig. 4:**
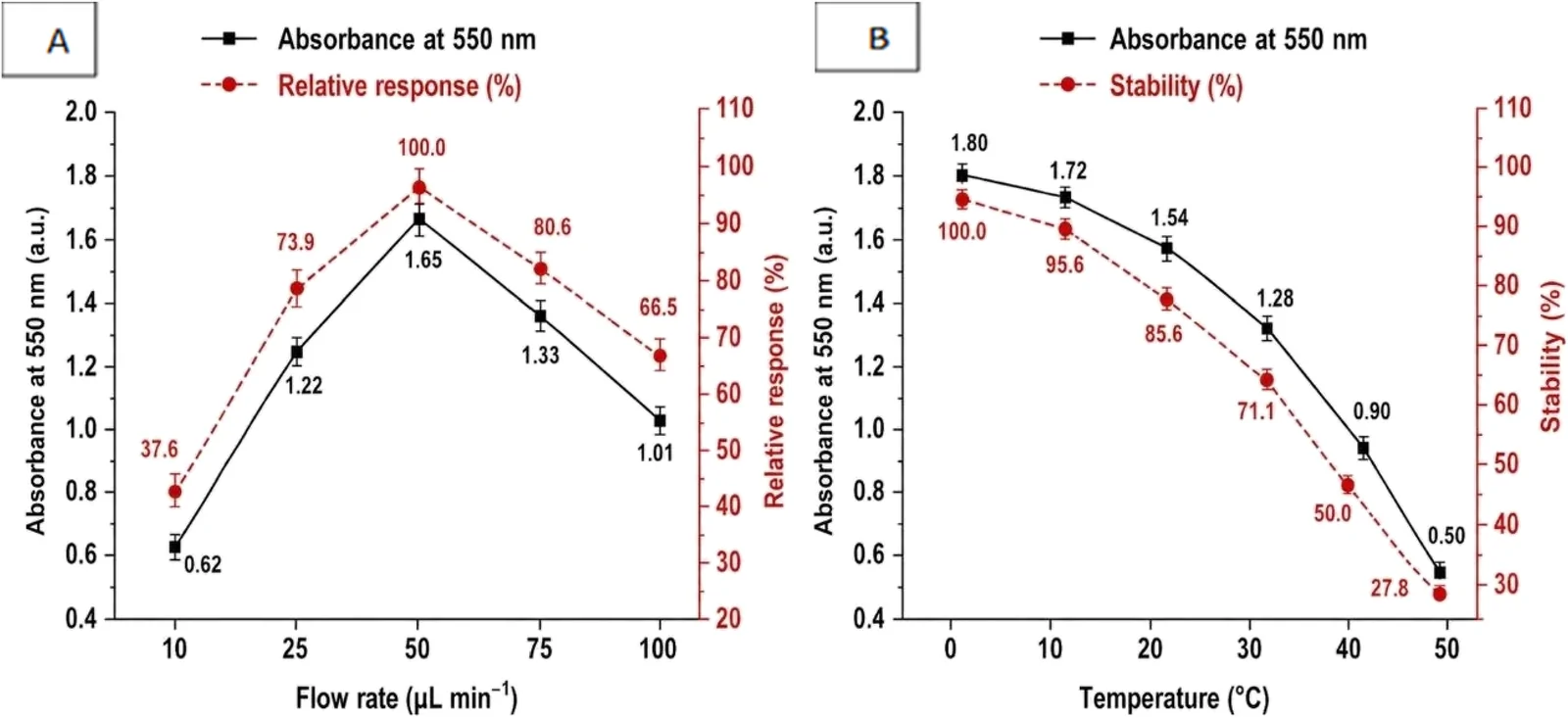
(A) Effect of flow rate and (B) effect of temperature on the absorbance response and stability of the nanocurcumin–l-arginine complex within the microfluidic sensing platform at 550 nm. Optimization was performed using 10 µmol per L nanocurcumin and 10 µmol per l-arginine. All experiments were performed in triplicate (*n* = 3), and the results are presented as mean ± standard deviation (SD).

### 4.2 The optimization of nanocurcumin concentration and operational performance of the microfluidic platform

The analytical performance of the proposed nanocurcumin-based microfluidic sensing platform was optimized by studying the effect of nanocurcumin concentration on the absorbance response at 550 nm. The absorbance and relative sensing response were found to increase with increasing nanocurcumin concentration, as shown in Fig. 5A which indicated increased formation of the nanocurcumin–arginine complex and improved optical signal generation, within the microfluidic channel. The highest analytical response was recorded at the concentration of 0.55 µM, which was used for all the subsequent experiments. When higher concentrations were used, a slight increase in the analytical signal was seen and the interaction sites were approaching saturation, so that additional nanocurcumin did not have a significant effect on the sensing performance. The overall contribution of optimized experimental parameters to the analytical performance of the sensing platform is provided in Fig. 5B. The concentration of nanocurcumin (24%), flow rate (22%), temperature (20%), carrier medium (18%) and pH (16%) were found to be the most influential parameters. The relatively even distribution of these contributions indicates that the sensing performance is controlled by several operating parameters, rather than one single controlling parameter. This behavior proves the optimized microfluidic platform is stable and reproducible analytical performance under the selected experimental conditions.

**Fig. 5:**
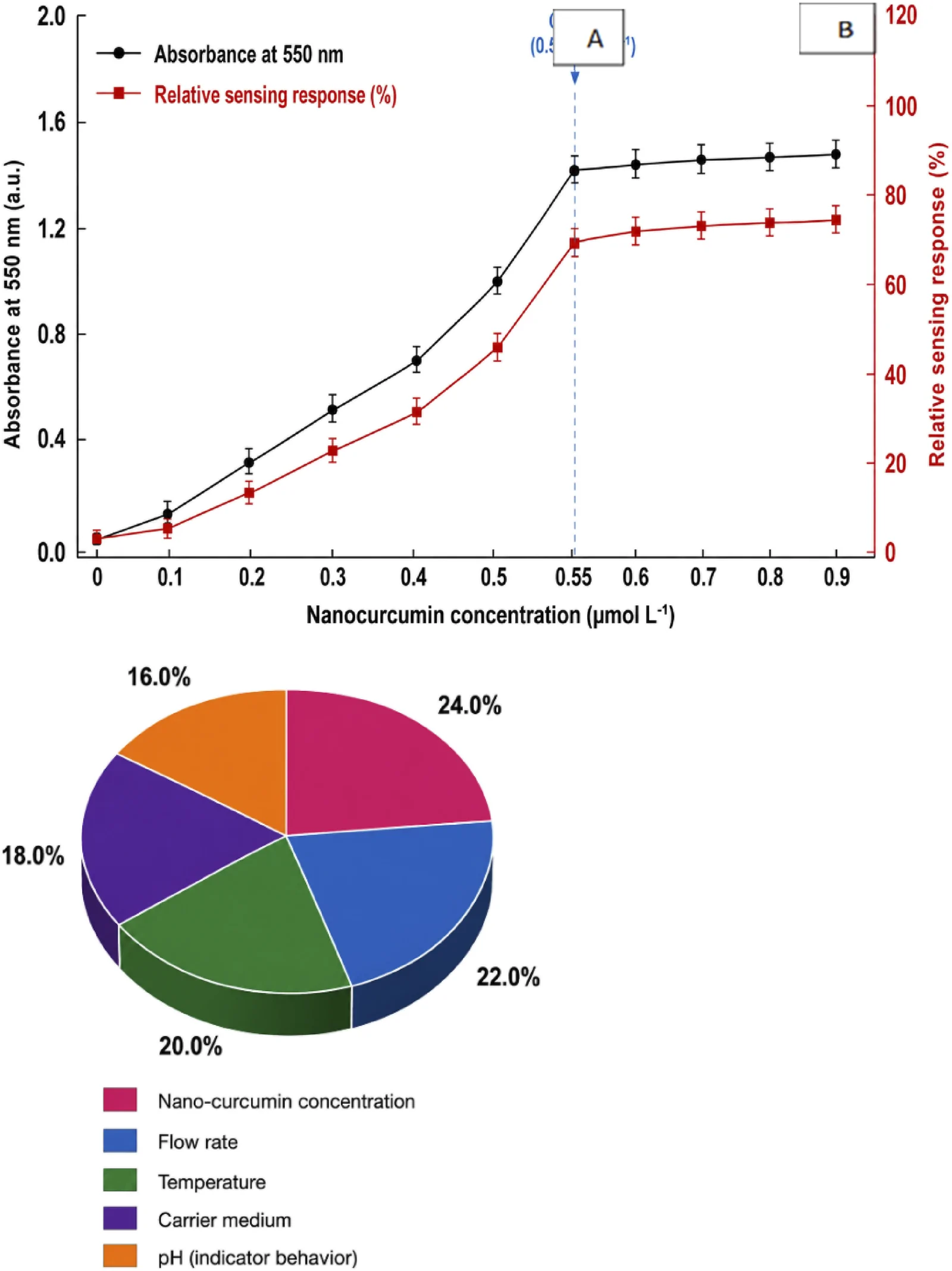
Optimization of the nanocurcumin-based microfluidic sensing platform. (A) Effect of nanocurcumin concentration on absorbance and relative sensing response. (B) Relative contribution of the optimized experimental parameters to the analytical performance at 550 nm.

### 4.3 Microfluidic absorbance response behavior of the nanocurcumin–arginine complex

The absorbance response profile of nano-curcumin, l-arginine and the nanocurcumin–arginine complex under the optimized microfluidic sensing conditions at 550 nm is shown in Fig. 6. As the concentration of nanocurcumin increased, the absorbance intensity also increased, which showed that the interaction efficiency was better, and the complex formation was more efficient in the microfluidic channel. The absorbance values of the nanocurcumin–arginine complex were significantly higher than those of nanocurcumin and l-arginine alone, indicating the successful formation of the nanocurcumin–arginine complex and the synergistic increase in the optical response. The maximum analytical response was found at 0.55 µmol L^−1^ nanocurcumin concentration, which is the optimum sensing condition for efficient signal generation and analytical sensitivity. At higher concentrations of nano-curcumin, a slight decrease in absorbance response was observed, which could be explained by partial signal saturation and reduced optical efficiency at higher concentrations of nano-curcumin. In general, the response profile obtained shows that the proposed nano-curcumin microfluidic platform is suitable for sensitive and stable colorimetric determination of l-arginine.

**Fig. 6:**
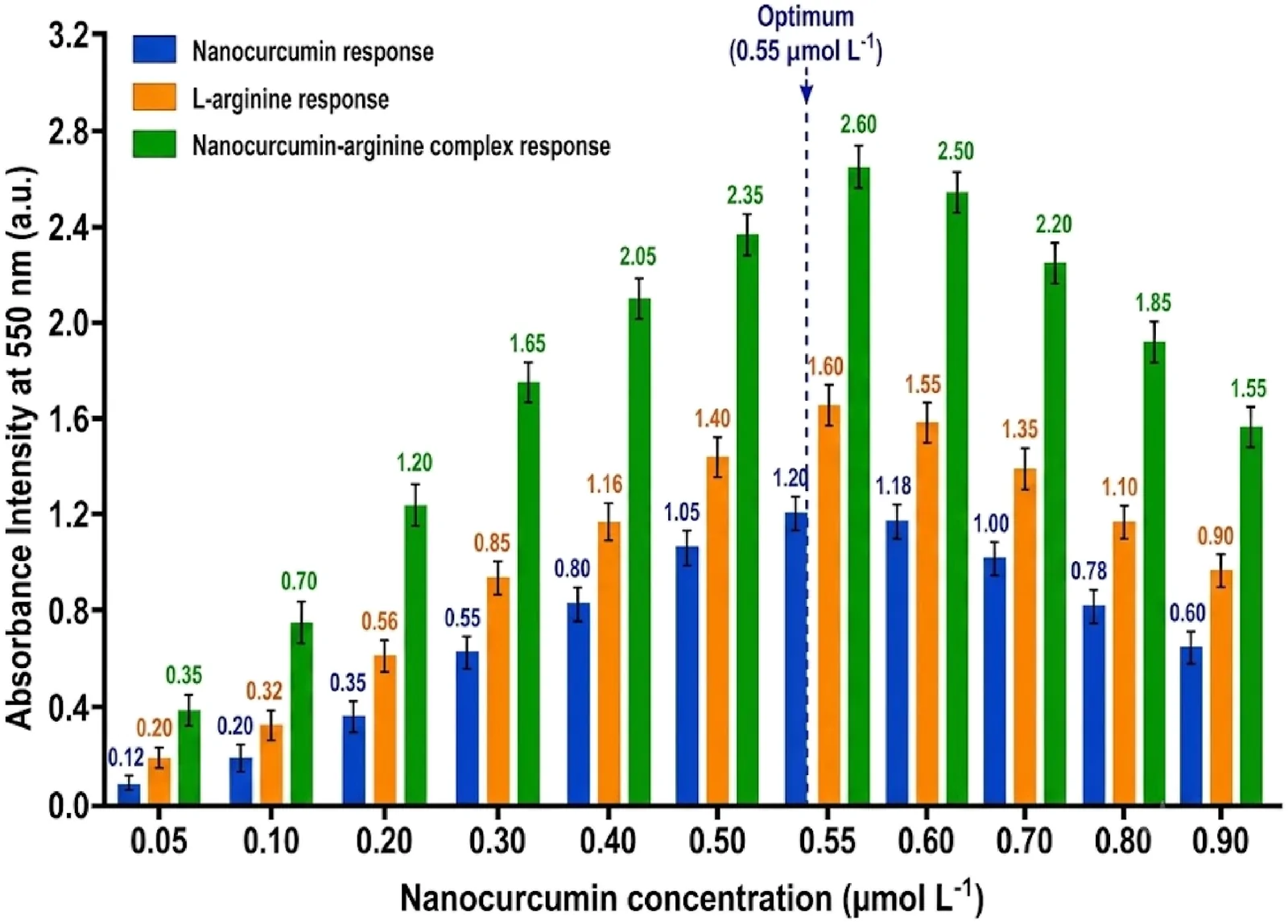
Absorbance response profile of nanocurcumin, l-arginine, and the nanocurcumin–arginine complex under optimized microfluidic sensing conditions at 550 nm.

### 4.4 Interpretation of Job's plot

The Job's plot exhibited a maximum absorbance at a nano-curcumin mole fraction of 0.33, confirming that the complex formed between nanocurcumin and l-arginine possesses a 2 : 1 (l-arginine : nanocurcumin) stoichiometric ratio. This result indicates that two molecules of l-arginine bind to one molecule of nanocurcumin, leading to the formation of the most stable colored complex. The observed stoichiometry supports the proposed sensing mechanism and explains the enhanced absorbance obtained under the optimized experimental conditions, as shown in Fig. 7 and Table 1.^25,30^

**Fig. 7:**
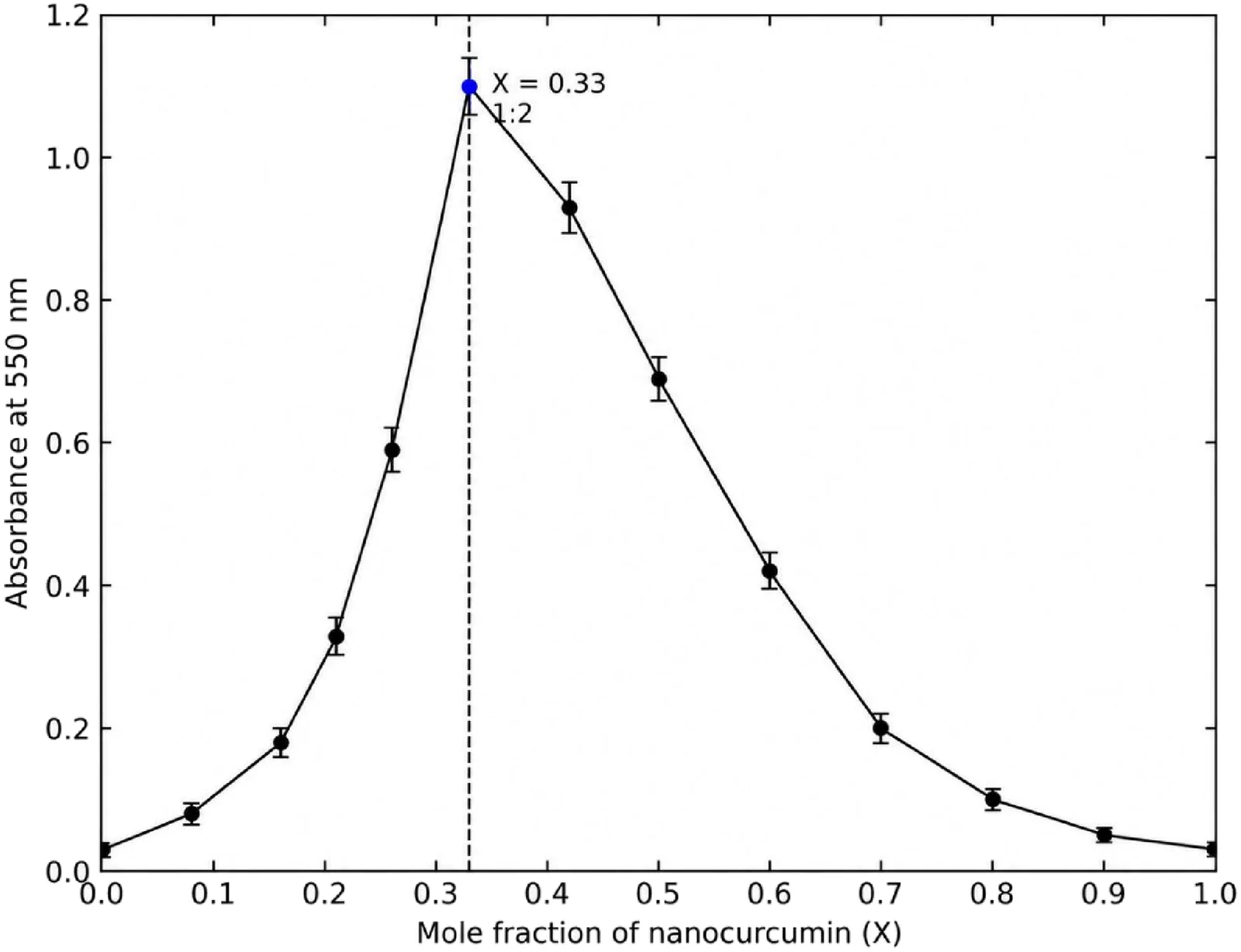
Job's plot illustrating the stoichiometric ratio of the nanocurcumin–l-arginine complex formation.

**Table 1:** Experimental absorbance values at 550 nm for Job's plot (continuous variation method) used to determine the stoichiometric ratio of the nanocurcumin–l-arginine complex

Mole fraction of nanocurcumin (*X*)	Absorbance *λ* = 550 nm
0.0	0.0311
0.1	0.0825
0.15	0.1838
0.2	0.3306
0.25	0.5926
**0.33**	**1.0984**
0.4	0.9385
0.5	0.6973
0.6	0.4219
0.7	0.2017
0.8	0.1016
0.9	0.0515
1.0	0.0394

### 4.5 Calibration performance of microfluidic nanocurcumin–l-arginine sensing systems

In Fig. 8 The microfluidic system demonstrated higher analytical sensitivity and better signal response with increasing l-arginine concentration, with a higher slope value and correlation coefficient (*R*^2^ = 0.9993) compared to the other system. As shown in Table 2, the results obtained indicate that the microfluidic sensing system is more efficient and reliable, and sensitive colorimetric detection of l-arginine than the conventional analytical approach. The calibration slope of the microfluidic platform (0.4000) was higher than the conventional UV-Vis method (0.2000), indicating higher analytical sensitivity. Each concentration level was measured in triplicate (*n* = 3). The results are presented as mean ± standard deviation (SD) at (*λ* = 550 nm). This enhancement is due to the efficient mixing, short diffusion path, and effective mass transfer in the microfluidic channels.

**Fig. 8:**
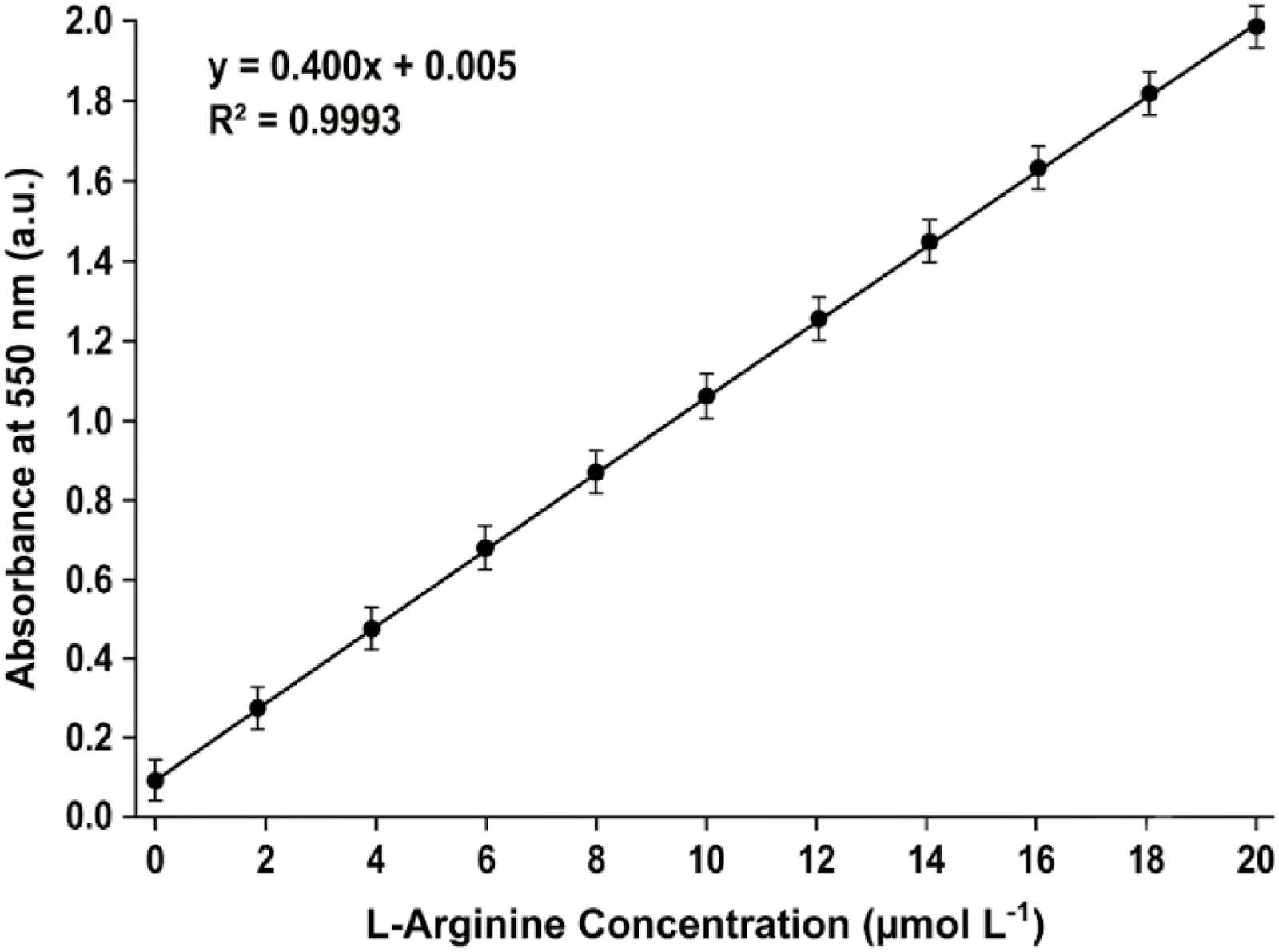
Calibration curve for l-arginine determination using the nanocurcumin microfluidic sensing platform. Each concentration level was measured in triplicate (*n* = 3). The results are presented as mean ± standard deviation (SD) at (*λ* = 550 nm).

**Table 2:** The analytical performance results of the nanocurcumin microfluidic sensing platform for l-arginine determination (*λ* = 550 nm)

Parameter	Nanocurcumin–arginine compound	Unit	Description
Detection wavelength	550	nm	Laser excitation wavelength
Linear range (LDR)	0–20	µmol L^−1^	Spiked concentration range
Calibration equation	*y* = 0.400*x* + 0.0050	—	*x*: concentration (µmol L^−1^), *y*: signal (a.u.)
Sensitivity (*m*)	0.400	a.u. µmol L^−1^	Slope of calibration curve
Intercept (*b*)	0.005	a.u.	Calibration intercept
Correlation coefficient (*R*^2^)	0.9993	—	Linearity of the sensor
Standard deviation of blank (*σ*)	0.000100	a.u	Noise level (*n* = 3)
Limit of detection (LOD)	0.825	nmol L^−1^	LOD = 3.3*σ*/*m*
Limit of quantification (LOQ)	2.5	nmol L^−1^	LOQ = 10*σ*/*m*
Repeatability (RSD%)	0.14	%	*n* = 3 at 5 µmol L^−1^
Recovery (%)	99.1–101.4	%	Accuracy of real sample analysis
*T*-Test result	4.303		Statistical evaluation
*F*-Test result (ANOVA)	19		Statistical evaluation
*P*-Value	*p* < 0.05		Statistical significance
Absorbance range	0.05–2	a.u.	Experimental response range

### 4.6 Selectivity and spectral overlap evaluation

Under optimized experimental conditions, the selectivity of the nanocurcumin-based microfluidic sensing platform for l-arginine was quantitatively evaluated by recording the full UV-Vis absorption spectra (400–700 nm). The concentration of l-arginine was set at 4.0 µmol L^−1^ (midpoint of the verified dynamic analytical range), and all co-existing interferents (metal ions, simple amino acids, organic metabolites and excipients) were tested at a 50-fold molar excess (200 µmol L^−1^).

Foreign species did not show any significant spectral overlap or absorption peak shift at *λ* = 550 nm when compared to the baseline absorption characteristics of free nanocurcumin as shown in Fig. 9. The l-arginine, on the other hand, caused a clear bathochromic shift with a strong absorption peak at 550 nm and a marked color change from yellow to reddish-orange. The high molecular recognition is due to the strong hydrogen bonding and electrostatic interactions between the guanidinium group of l-arginine and the oxygen-containing functional groups of nanocurcumin, which significantly increases the intramolecular charge transfer (ICT).

**Fig. 9:**
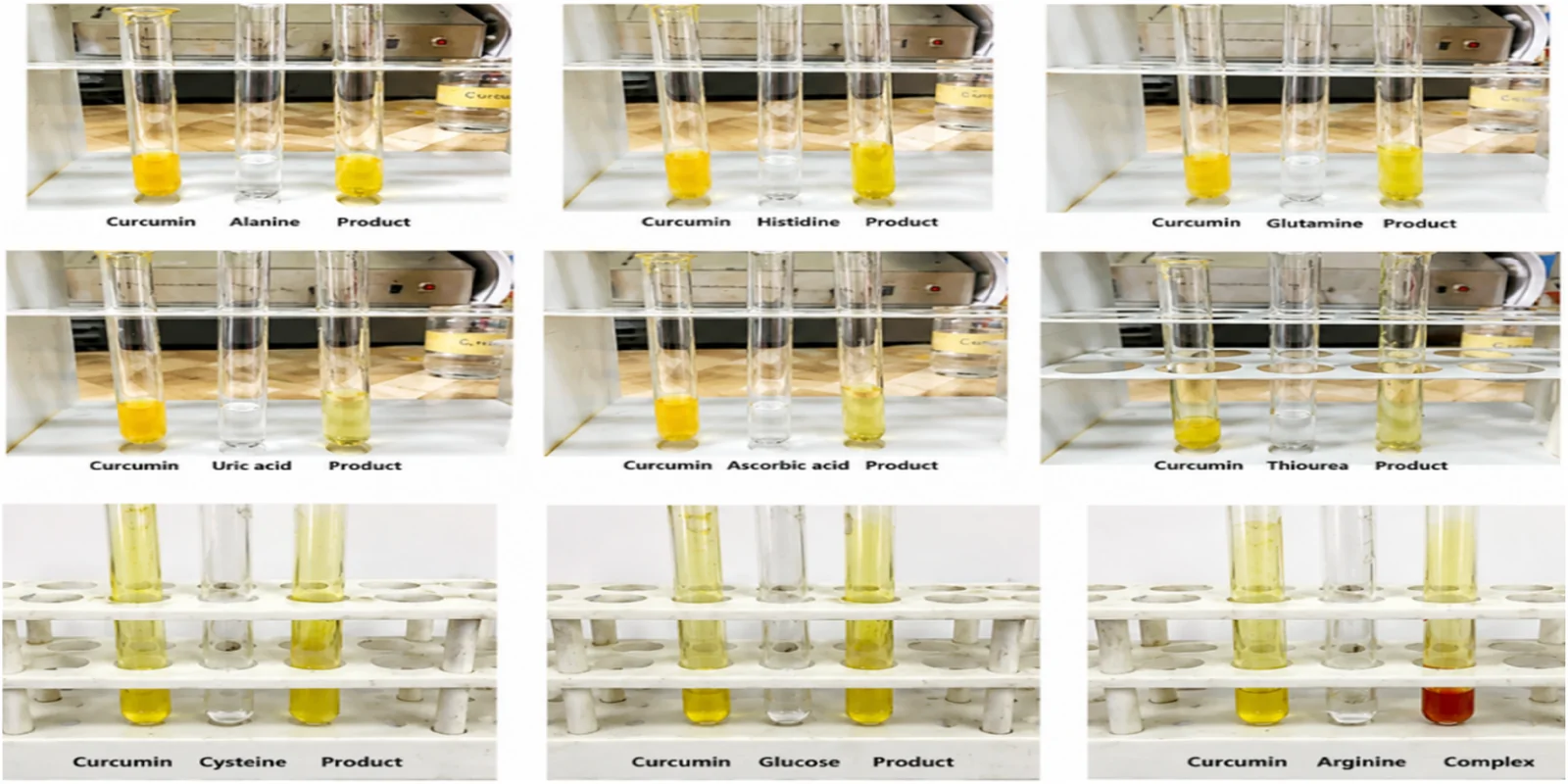
Selectivity study of nanocurcumin toward various analytes, showing a selective bathochromic shift and enhanced absorbance for the nanocurcumin–l-arginine complex (*λ*_max_ = 550 nm).

### 4.7 Quantitative selectivity discussion

The absorbance response (*λ*_max_ = 550 nm) of l-arginine (4.0 µmol L^−1^) was compared with all co-existing species at 200 µmol L^−1^ (50-fold molar excess) under the same conditions (Table 3 and Fig. 10). The absorbance response of all foreign species was less than 4.5% of the response to l-arginine alone (Δ*E* < 0.05 a.u.). The selectivity factors (*K*_sel_ > 22.0) calculated indicate excellent discrimination ability and anti-interference ability of the nanocurcumin platform for l-arginine in complex matrices.
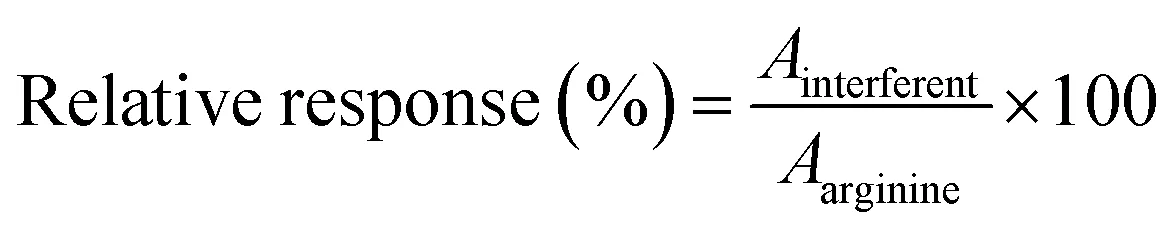

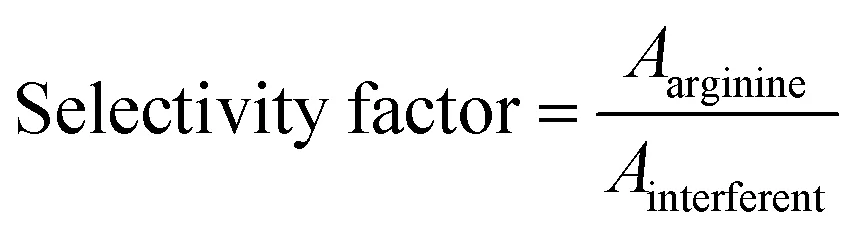


**Table 3:** Quantitative selectivity and interference evaluation of the nanocurcumin platform toward l-arginine (4.0 µmol L^−1^) in the presence of foreign species (200 µmol L^−1^, 50-fold excess) at *λ* = 550 nm (*n* = 3)

Interferent	Concentration (µmol L^−1^)	Absorbance at 550 nm (a.u.)	Relative response (%)	Selectivity factor	Approx. Δ*E*
Ag^+^	200	0.03	2.7	36.7	4.0
Zn^2+^	200	0.02	1.8	55.0	6.2
Mg^2+^	200	0.02	1.8	55.0	10–8
Bi^3+^	200	0.03	2.7	36.7	7.9
Cu^2+^	200	0.02	1.8	55.0	2.6
Fe^3+^	200	0.05	4.5	22.0	15.2
Ca^2+^	200	0.03	2.7	36.7	10–8
Alanine	200	0.04	3.6	27.5	5–3
Urea	200	0.03	2.7	36.7	7–5
Glycine	200	0.03	2.7	36.7	5–3
Uric acid	200	0.03	2.7	36.7	6–4
l-Arginine	4.0	1.10	100	1.00	≈45–50 (reference to curcumin)

**Fig. 10:**
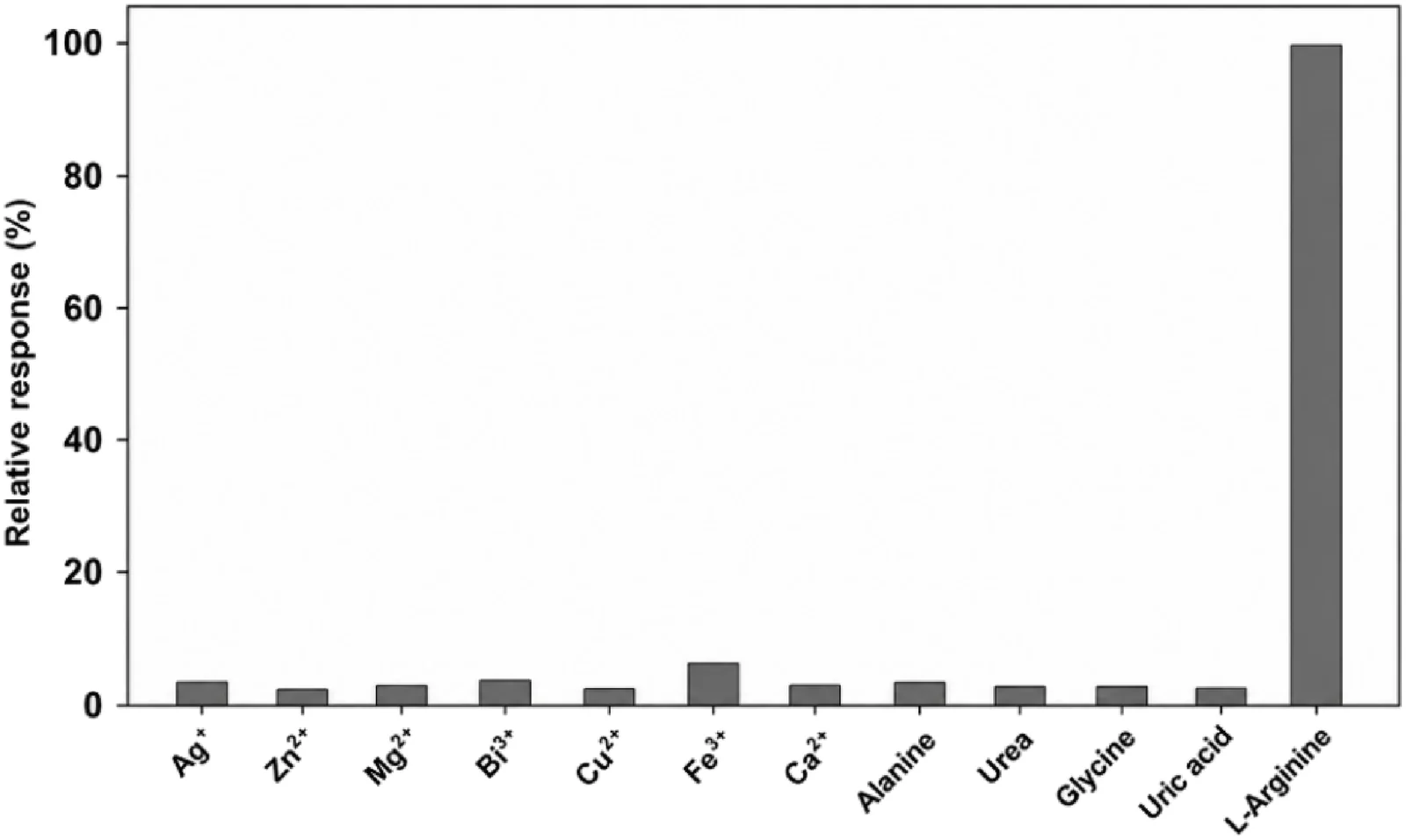
Relative response (%) of the proposed nanocurcumin toward l-arginine and various potential interfering species under identical experimental conditions. The response of l-arginine was normalized to 100%.

### 4.8 Interference study

The selectivity and matrix tolerance of the proposed nanocurcumin-based microfluidic sensing platform for l-arginine determination were thoroughly evaluated under optimized experimental conditions. Structural analogy (amino acids with similar functional groups, such as histidine, glycine, and alanine) and matrix relevancy in biological and pharmaceutical samples (nitrogenous metabolites such as urea and uric acid, and common inorganic ions) were used to select potential co-existing species.

To test the interference threshold, all foreign species were introduced at much higher molar excess ratios (10 : 1 and 20 : 1) compared to l-arginine. The analytical response was followed spectrophotometrically at *λ* = 550 nm and matrix effects were assessed by percentage recovery (%) and relative standard deviation (RSD%). Excellent recoveries (97.5% to 101.2%) and low RSD values (≤1.5%) were obtained for all species tested (Table 4). The results validate that high levels of structurally similar compounds and matrix co-constituents do not interfere significantly, thus demonstrating the high selectivity and reliability of the proposed sensing platform.

**Table 4:** Interference study of potential foreign species on l-arginine determination at elevated concentration ratios (*n* = 3)

Species	Concentration ratio (interferent : arginine)	Recovery (%)	RSD (%)
Ag^+^	10 : 1	98.5	1.2
Pb^2+^	10 : 1	99.1	1.0
Hg(ii)	10 : 1	97.8	1.5
Bi^3+^	10 : 1	101.2	1.3
Cd^2+^	10 : 1	99.4	1.1
Histidine	10 : 1	100.5	0.9
Glycine	20 : 1	98.9	1.2
Alanine	20 : 1	99.1	1.0
Urea	20 : 1	101.0	1.0
Uric acid	20 : 1	97.5	1.4

### 4.9 Application to real samples

The proposed nanocurcumin-based microfluidic sensing platform was applied to the determination of l-arginine in pharmaceutical capsule, human serum and urine samples to evaluate its practical applicability. The standard-addition method was used to reduce the matrix interference that is inherent in biological and pharmaceutical media. Standard-addition calibration plots incorporating all experimental observations (0–10 µmol L^−1^) were constructed individually for each matrix, as shown in Fig. 11. The linear regression equations, verified linear working ranges and analytical performance parameters are summarized in Table 5. The verified linear working ranges were established as 0–8 µmol L^−1^ for capsule samples, 0–4 µmol L^−1^ for serum samples, and 0–6 µmol L^−1^ for urine samples. The calibration plots showed good linearity within these linear ranges, with correlation coefficients (*R*^2^) of 0.9993, 0.9979, and 0.9921 for capsules, serum, and urine, respectively. The data points falling outside these ranges were retained as experimental observations, but were not used for quantitative regression calculations due to response saturation and non-linear deviations at higher concentrations caused by the matrix.

**Fig. 11:**
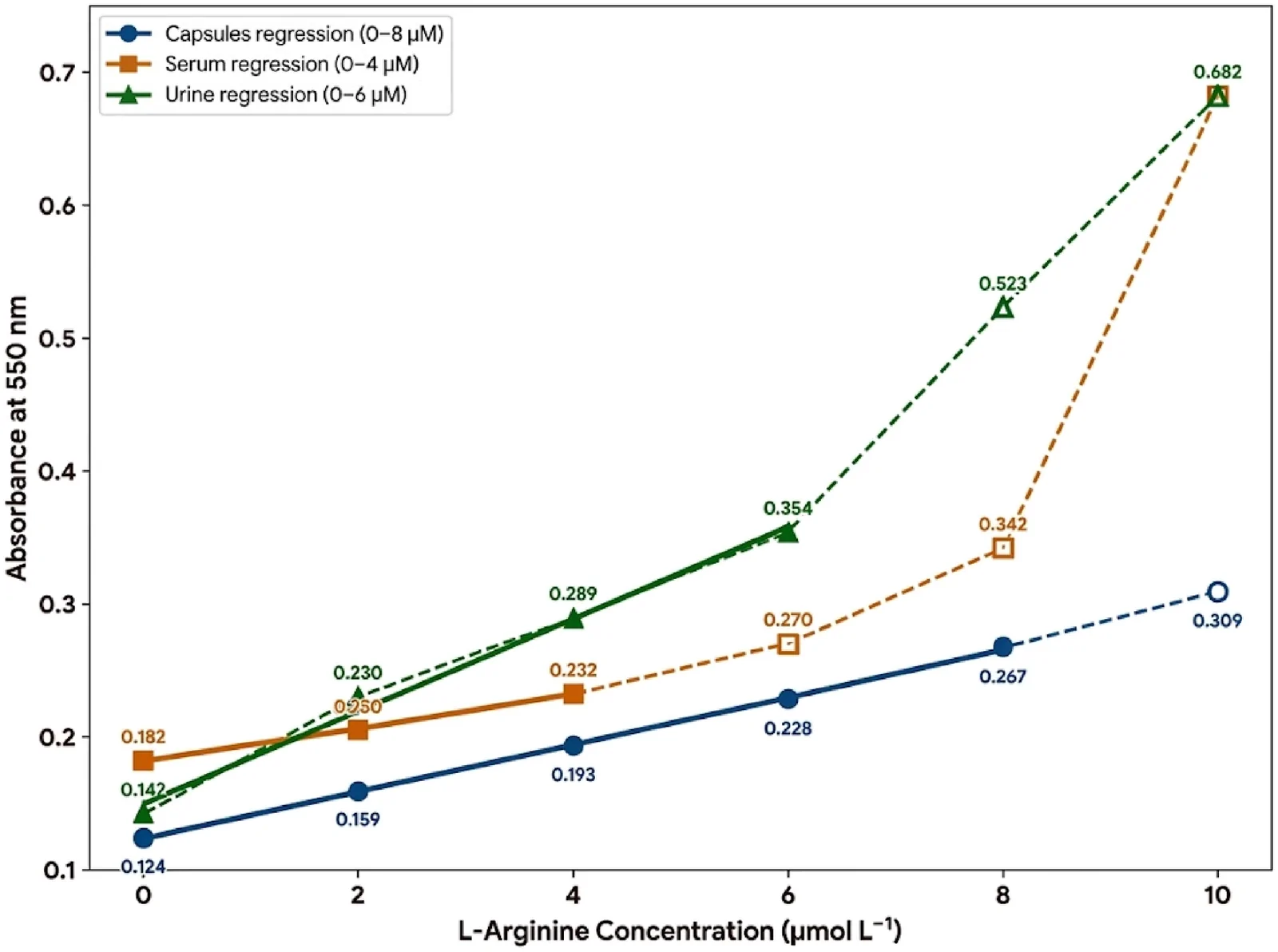
Standard-addition calibration plots for the determination of l-arginine in capsule, serum, and urine samples using microfluidic spectrophotometry at *λ* = 550 nm. Solid lines represent the linear regression fitted within the verified linear working ranges (0–8 µmol L^−1^ for capsules, 0–4 µmol L^−1^ for serum, and 0–6 µmol L^−1^ for urine). Solid markers indicate data points included in the quantitative linear regression calculations, whereas open markers represent experimental observations exhibiting matrix-induced non-linear.

**Table 5:** Analytical performance of the nanocurcumin-based microfluidic sensing platform for the determination of l-arginine in pharmaceutical and biological samples using using standard-addition calibration (*λ* = 550 nm)

Parameter	In capsules samples	In serum samples	In urine samples	Unit	Description
Detection wavelength	550	550	550	nm	Analytical wavelength
Linear working range	0–8	0–4	0–6	µmol L^−1^	Verified linear standard-addition range
Standard-addition calibration equation	*y* = 0.01775*x* + 0.12320	*y* = 0.01250*x* + 0.18133	y= 0.03475*x* + 0.14950	—	*x*: added l-arginine concentration (µmol L^−1^); *y*: signal (a.u.)
Slope (*m*)	0.01775	0.01250	0.03475	a.u. µmol L^−1^	Slope of standard-addition plot
Intercept (*b*)	0.12320	0.18133	0.14950	a.u.	Standard-addition intercept
Correlation coefficient (*R*^2^)	0.9993	0.9979	0.9921	—	Linearity within working range
Standard deviation of blank (*σ*)	0.00985	0.01240	0.00920	a.u	Blank standard deviation (*n* = 3)
Limit of detection (LOD)	1831.3	3273.6	873.7	nmol L^−1^	LOD = 3.3*σ*/*m*
Limit of quantification (LOQ)	5549.3	9920.0	2647.5	nmol L^−1^	LOQ = 10*σ*/*m*
Repeatability (RSD%)	0.87	0.31	0.54	%	*n* = 3 at 6 µmol L^−1^
Recovery (%)	98.5–101.8	95.2–99.4	94.8–98.7	%	Accuracy of real-sample analysis
*T*-Test result	4.303	4.303	4.303		Statistical evaluation
*F*-Test result (ANOVA)	19	19	19		Statistical evaluation
*P*-Value	*p* < 0.05	*p* < 0.05	*p* < 0.05		Statistical significance
Absorbance range	0.124–0.309	0.182–0.682	0.142–0.682	a.u.	Total experimental response range (0–10 µmol L^−1^)

The slight differences in slopes and linear response limits of the investigated matrices are directly related to the differences in matrix complexity and the different matrix effects on the colorimetric interaction between nanocurcumin and l-arginine.

The recoveries obtained were in the range of 94.8% to 101.8% and the relative standard deviation (RSD) values were in the range of 0.31% to 0.87%, which indicated good accuracy and precision. The results showed that the standard-addition method was able to compensate for the possible matrix effect and the proposed microfluidic platform was successfully applied to the determination of l-arginine in pharmaceutical and biological samples.

### 4.10 Accuracy and recovery studies

Spiking recovery assays were performed at three concentration levels (*n* = 3) within the linear range of each sample type to assess accuracy. The baseline endogenous l-arginine concentrations determined prior to spiking were 6.94 µmol L^−1^ for diluted capsule solutions, 14.50 µmol L^−1^ for diluted serum (145.0 µmol L^−1^ undiluted), and 4.30 µmol L^−1^ for diluted urine (86.0 µmol L^−1^ undiluted). Excellent recoveries between 94.8% and 101.7% with RSD < 1.0% were achieved, proving the accuracy and reliability of the sensing platform in complex matrices, are summarized in Table 6.
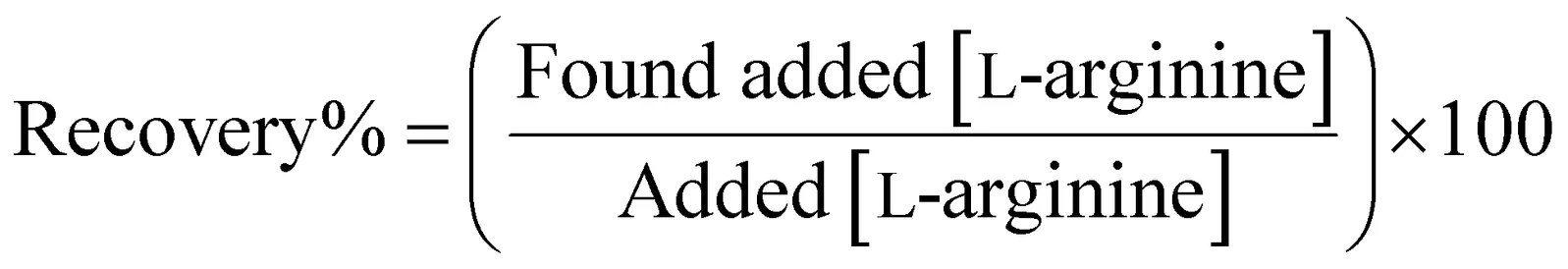

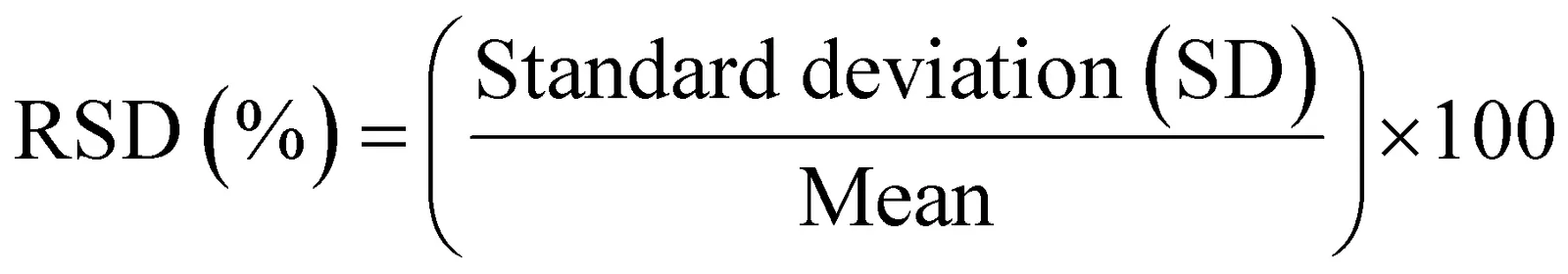


**Table 6:** Recovery results for l-arginine determination in real samples using the proposed microfluidic method

Sample	Endogenous [l-arginine] (µmol L^−1^)	Added [l-arginine] (µmol L^−1^)	Found [l-arginine] (µmol L^−1^)	RSD (%) (*n* = 3)	Recovery (%)
Capsules	6.94	1.00	0.98	98.0	0.87
Capsules	6.94	3.00	3.05	101.67	0.62
Capsules	6.94	5.00	4.93	98.60	0.45
Serum	14.50	0.50	0.48	96.0	0.31
Serum	14.50	1.50	1.43	95.33	0.42
Serum	14.50	2.50	2.48	99.20	0.28
Urine	4.30	1.00	0.96	96.00	0.54
Urine	4.30	2.00	1.97	98.50	0.38
Urine	4.30	4.00	3.89	97.25	0.41

## 5. Comprehensive greenness and whiteness assessment of the microfluidic platform

Five complementary assessment frameworks were applied (Fig. 12): GAPI, AGREE, NACaWa, CaFRI/Footprint and WAC/RGB 12, to quantitatively and holistically determine the environmental sustainability and operational efficiency of the proposed nanocurcumin-based microfluidic method for l-arginine determination. All software algorithms have detailed input parameters as described in Table S2.^31^

**Fig. 12:**
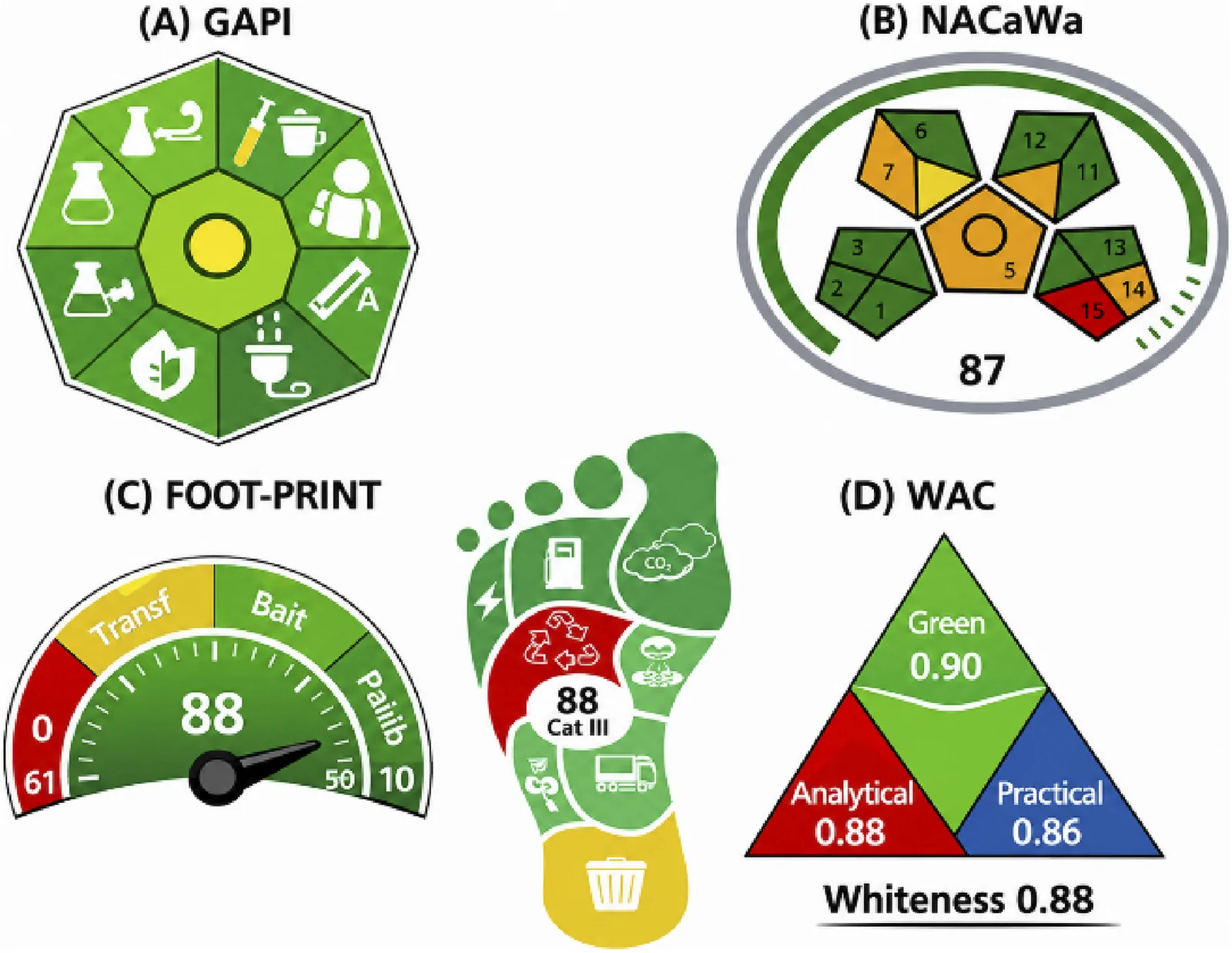
Greenness and whiteness assessment of the proposed microfluidic platform for l-arginine determination using (A) GAPI, (B) NACaWa, (C) footprint (CaFRI), and (D) White Analytical Chemistry (WAC) tools.

• GAPI (Fig. 12A): the GAPI pictogram evaluates 15 operational criteria that include sample collection, preparation, solvents/reagents, and instrumentation/waste generation. The proposed method has a predominantly green profile because of the following: (i) offline sample preparation without use of organic solvents, (ii) benign aqueous medium (distilled water) that eliminates organic solvents, (iii) low volume of sample and reagent (less than (<50 µL), and (iv) low energy consumption <0.1 kWh and low generation of hazardous waste (<1 mL per nalysis).

• AGREE assessment: the AGREE metric (score = 0.88) combines the 12 principles of Green Analytical Chemistry. The microfluidic platform had high sub-scores compared to batch spectrophotometric methodologies, in terms of miniaturization, direct inline colorimetric readout, high sample throughput, and absolute avoidance of hazardous chemical reagents.

• NACaWa evaluation (Fig. 12B): the semi-quantitative NACaWa framework resulted in a total score of 8.7/10.0 indicating low ecological and occupational risk. The minor penalty was due to the synthesis step of nanocurcumin only, which is compensated by its subsequent dilution and aqueous stability.

• CaFRI/footprint index (Fig. 12C): the carbon footprint and resource index achieved a high sustainability rating of 88.0/100, indicating very low energy consumption, low waste treatment requirements and low exposure to chemicals during normal operation.

• White analytical chemistry WAC/RGB 12 (Fig. 12D): WAC model is a synthesis of three basic dimensions, namely red (analytical performance: LOD, linearity, accuracy; score = 0.88), green (environmental & ecological safety; score = 0.90) and blue (practicality & cost-efficiency; score = 0.89). The overall whiteness score of 89.0% indicates that the proposed sensing platform is not only environmentally friendly but also analytically sound and suitable for regular quality control.

## 6. Comparison between the conventional batch method and the proposed

The conventional batch procedure is directly compared with the proposed nanocurcumin-based microfluidic sensing platform in Fig. 13. The microfluidic platform was found to have a lower detection limit, significantly shorter analysis time, lower reagent usage and significantly less waste compared to the conventional batch method. The improvements highlight the analytical efficiency and environmental friendliness of the proposed platform, which makes it applicable to routine and green analytical applications. A detailed comparison with the previously reported methods is given in Table 7.

**Fig. 13:**
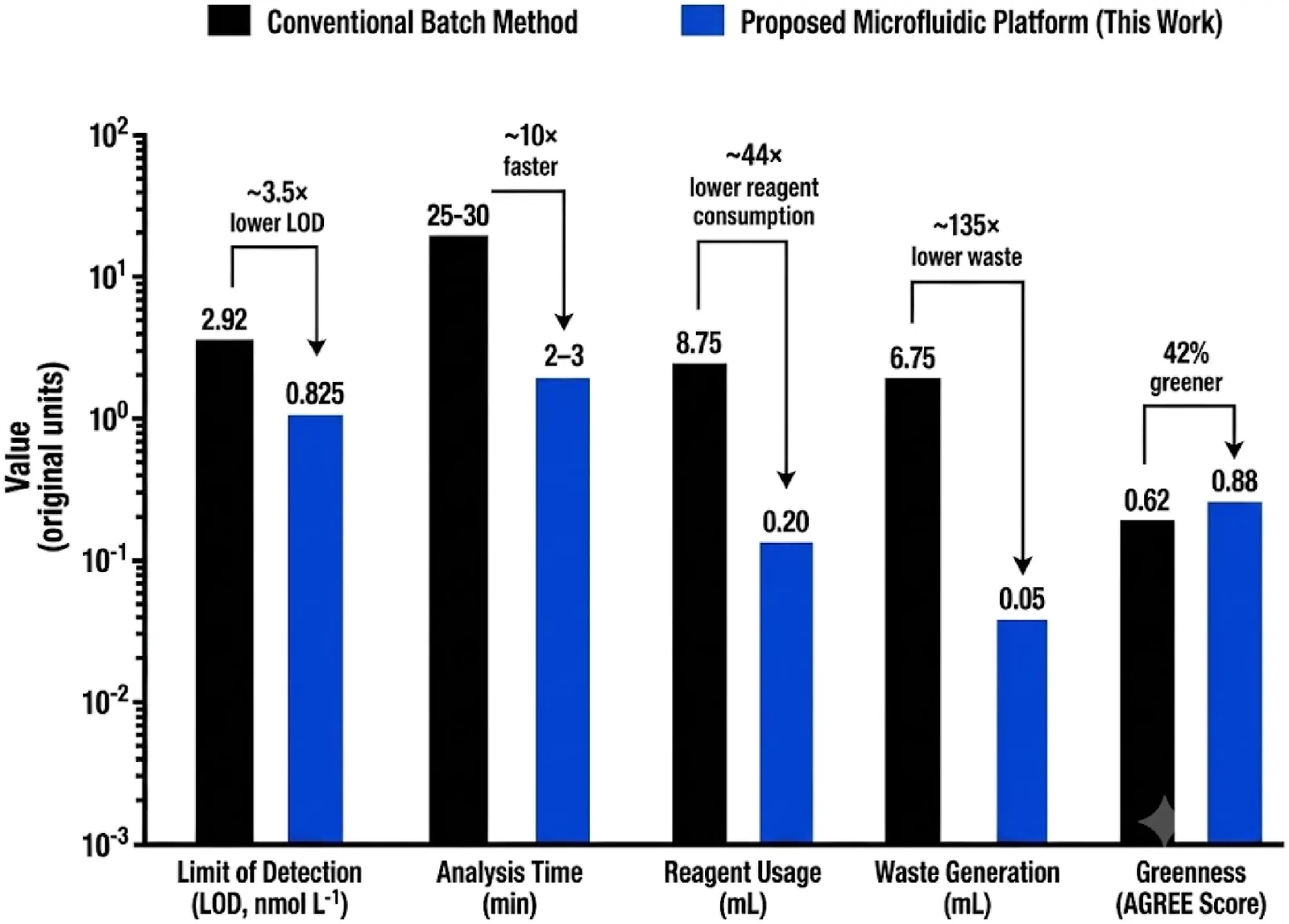
Comparative between conventional batch method and the proposed microfluidic platform for l-arginine determination.

**Table 7:** Comparison of the analytical performance of the proposed nanocurcumin colorimetric sensor with recently reported methods for l-arginine determination^a^

Method	Detection technique	Linear range	LOD	Recovery (%)	RSD (%)	AGREE score	Main limitation	Ref.
Azo nano-materials biosensor	UV-Vis colorimetric nanosensor	5–50 µM	NR	NR	NR	NR	Uses an azo nanomaterial system requiring synthesis and nanomaterial preparation; the study also focuses on biosensor/cytotoxicity evaluation rather than a simple naked-eye colorimetric platform	32
Citrate-capped AuNPs aggregation	Visual + UV-Vis	0.08–13.2 µM	16 nM	NR	NR	NR	Requires citrate-capped AuNPs and controlled pH; nanoparticle aggregation can be affected by matrix composition and ionic strength	33
4-Amino nicotinic acid assembled gold nanoparticles (ANA-AuNPs)	Colorimetric/UV-visible spectrophotometry	0.001–100 µM	7.2 nM	NR	NR	NR	Requires AuNP synthesis/functionalization and is not exclusively selective for l-arginine, as the platform also responds to His, Met and Trp	34
UV-Vis (diacetyl monoxime)	Spectrophotometric	100–5000 µM	15 × 10^3^ nM	94–100	1.8–3.5	NR	Requires chromatographic instrumentation and sample preparation; therefore, it is less suitable for rapid, simple, naked-eye screening. The method also showed reduced specificity/accuracy for arginine in complex corn-steep-liquor medium	35
ThiCS (thiazole-based azo chemosensor)	Colorimetric/fluorescence	NR	NR	NR	NR	NR	Requires aqueous–acetonitrile medium and fluorescence measurements for dual-mode detection	36
AgNP-based colorimetric sensor	Nanoparticle UV-Vis probe	8 × 10^−2^–132 × 10^−1^ µM	15 × 10^1^ nM	94–101	1.2–2.5	NR	Requires AgNP preparation and UV-Vis measurement; the reported Arg linear range is relatively narrow (0.5–4.5 µM)	37
ArgO–nanozyme biosensor	Amperometric biosensor	600–10000 M	5 × 10^3^ nM	97.1–102.5	2.4	0.76	Requires enzyme immobilization and electrode-based amperometric measurements; the multicomponent bioselective layer increases fabrication complexity	38
Fe_3_O_4_@SiO_2_–Ag nanosensor	Colorimetric nanosensor	0.03–0.06 µM	2.56 × 10^3^ nM	98.2–101.8	1.9	0.81	Requires a relatively complex Fe_3_O_4_@SiO_2_@Ag nanocomposite	39
Vanadium-docked porphyrinic COF nanozyme	Colorimetric nanozyme sensor	NR	4 × 10^3^ nM	97.5–103.1	1.7	0.84	Requires a photo-responsive vanadium-docked porphyrinic COF nanozyme and light irradiation, increasing material and operational complexity	40
AuNP apta-nanosensor	Colorimetric apta-sensor	NR	38 × 10^2^ nM	98.4–102.7	1.5	0.87	Requires aptamer-functionalized AuNPs and salt-induced aggregation; matrix effects may affect the colorimetric response	8
Benzothiazole–azo sensor (BTAN)	UV-Vis/^1^H NMR colorimetric sensor	NR	7 × 10^3^ nM	91	1.79	NR	Although it supports aqueous and solid-state sensing, implementation in hydrogel/film or paper-strip formats requires sensor fabrication and immobilization steps	41
TSPP-TA dual-signal probe	Colorimetric/fluorescence sensor	NR	35 × 10^2^ nM	98.8–103.5	1.3	0.86	Requires a dual-signal colorimetric/fluorescence platform and fluorescence instrumentation, making the system less straightforward than a simple colorimetric readout	42
Smartphone-assisted colorimetric probe	Colorimetric/ratio smartphone sensing	0–30 µM	57.91 nM	97.8–102.6	1.2–3.5	NR	Smartphone-assisted detection requires image acquisition and computational/image-processing procedures in addition to the chemical sensing step	43
			131.5 nM					
Conductometric biosensor	Conductometric biosensing	0–280 µM	5 × 10^3^ nM	96.5–101.8	1.5–4.0	NR	Requires enzyme co-immobilization (arginase/urease) on a conductometric electrode, making fabrication more complex than direct solution-phase colorimetry	44
HTPC colorimetric-fluorometric probe	UV-Vis & fluorescence	50–500 µM	2.77 × 10^3^ nM	95–101	1.0–2.2	0.74	Detects both lysine and arginine rather than being exclusively selective for l-arginine; the colorimetric	45
Graphene oxide–fluorescein sensor	Fluorescence turn-on/colorimetric sensing	1–100 µM	100 nM	98.2–103.1	1.1–2.8	NR	Requires fluorescence instrumentation for the highest sensitivity; naked-eye detection has a substantially	46
PMMA/paper hybrid chip	Microfluidic colorimetric platform	6.8 × 10^−3^–100 µ M	225 nM	—	—	—	Requires fluorescence instrumentation for the highest sensitivity; naked-eye detection has a substantially higher LOD compared to microfluidic spectrophotometric methods	47
Proposed method	Nanocurcumin microfluidic colorimetric sensor	0–20 µM	0.825 nM	99.1–101.4	0.14	0.88	Requires fluorescence instrumentation for the highest sensitivity; naked-eye detection has a substantially lower resolution at nanomolar levels	This work

a AGREE values for reference methods are cited directly from their respective original published studies. “NR” indicates that an AGREE metric evaluation was not reported in the original publication.

To our knowledge, only one microfluidic-based colorimetric platform has been reported for the determination of l-arginine using a PMMA/paper hybrid microfluidic chip with gold nanoparticles.^47^ The present study, on the other hand, presents the first microfluidic platform based on nanocurcumin for the selective UV-visible colorimetric determination of l-arginine. The proposed platform offers significantly better analytical performance than the previously reported microfluidic method, such as a significantly lower detection limit, excellent precision, satisfactory recovery, and enhanced environmental sustainability in line with the principles of green analytical chemistry.

## 7. Conclusion

A novel nanocurcumin-based colorimetric microfluidic sensing platform was successfully developed for selective determination of l-arginine. The sensing mechanism is attributed to the formation of a supramolecular nanocurcumin–arginine assembly stabilized by hydrogen bonding, electrostatic interactions and cation–π interactions, which results in the appearance of a characteristic absorption band at 550 nm and a distinct color transition from yellow to red. The combination of nanocurcumin and microfluidic technology resulted in a dramatic improvement in the analytical performance, as it increased the mixing efficiency, mass transfer and signal generation, while also decreasing the amount of reagents used and waste produced. The proposed platform exhibited good linearity, high sensitivity, low detection limits, good repeatability and good selectivity for l-arginine in the presence of potentially interfering substances under optimized operating conditions. The feasibility of the sensing platform was demonstrated by the successful analysis of pharmaceutical formulations and biological samples, with good recovery values and low matrix effects. In addition, greenness and whiteness evaluations showed the excellent environmental sustainability of the proposed method, which is suitable for the requirements of modern green analytical chemistry. In general, the developed nanocurcumin microfluidic platform is a simple, rapid, economical, and environmentally friendly alternative to the traditional analytical methods used for the determination of l-arginine. The proposed sensing strategy has great potential for future applications in clinical diagnostics, pharmaceutical analysis, environmental monitoring, and portable point-of-care analytical systems.

## 8. Future work

Further research is needed to optimize the architecture of the microchannels, to incorporate automated sample handling, to investigate other nanostructured curcumin derivatives, and to create portable, smartphone-based detection systems. The developments could further improve the analytical sensitivity, robustness and real-time monitoring of biomedical and environmental sensing applications.

## Author contributions

Wafaa Waleed Al-Qaysi: methodology, formal analysis, writing – review & editing, Statistical analysis, Statistical analysis, validation. Rawaa A. Faris: methodology, conceptualization, experimental design, validation, investigation. Alyaa Khider Abbas: methodology, conceptualization, investigation.

## Conflicts of interest

The authors declare that they have no competing financial or non-financial interests.

## Supplementary Material

RA-OLF-D6RA06294F-s001

## Data Availability

The data supporting the findings of this study, including the raw absorbance data, calibration curves, Job's plot data, FTIR and UV-Vis spectral data, and recovery/validation results, are available from the corresponding author upon reasonable request. Supplementary information (SI): characterization of curcumin (SEM, AFM, UV-vis). See DOI: https://doi.org/10.1039/d6ra06294f.
