# Supplementary material for: Detection of l-arginine
Source: RSC Adv. 2026 Nov 5. Online ahead of print. doi: 10.1039/d6ra06294f (PMC13737592; doi:10.1039/d6ra06294f)
Supplement: Supplementary file 1 [file RA-OLF-D6RA06294F-s001.pdf]

Supplementary Information (SI) for RSC Advances.
This journal is © The Royal Society of Chemistry

A Nanocurcumin-Enabled Microfluidic Platform for Selective Colorimetric Detection of L-Arginine

Wafaa Waleed Al-Qaysi*[1], Rawaa A. Faris[2], & Alyaa Khider Abbas[3]

[1,3]Department of Chemistry, College of Science, University of Baghdad, Baghdad, Iraq; [2] Institute of Laser for Postgraduate Studies, University of Baghdad, Baghdad, Iraq

wafa.w@sc.uobaghdad.edu.iq, rawaa@ilps.uobaghdad.edu.iq, & alyaa.abbas@sc.uobaghdad.edu.iq

iD https://orcid.org/0000-0003-4559-3041, 0000-0003-0181-5837, & 0000-0002-8400-0926

2.4 Characterization Data of nanocurcumin

1. SEM

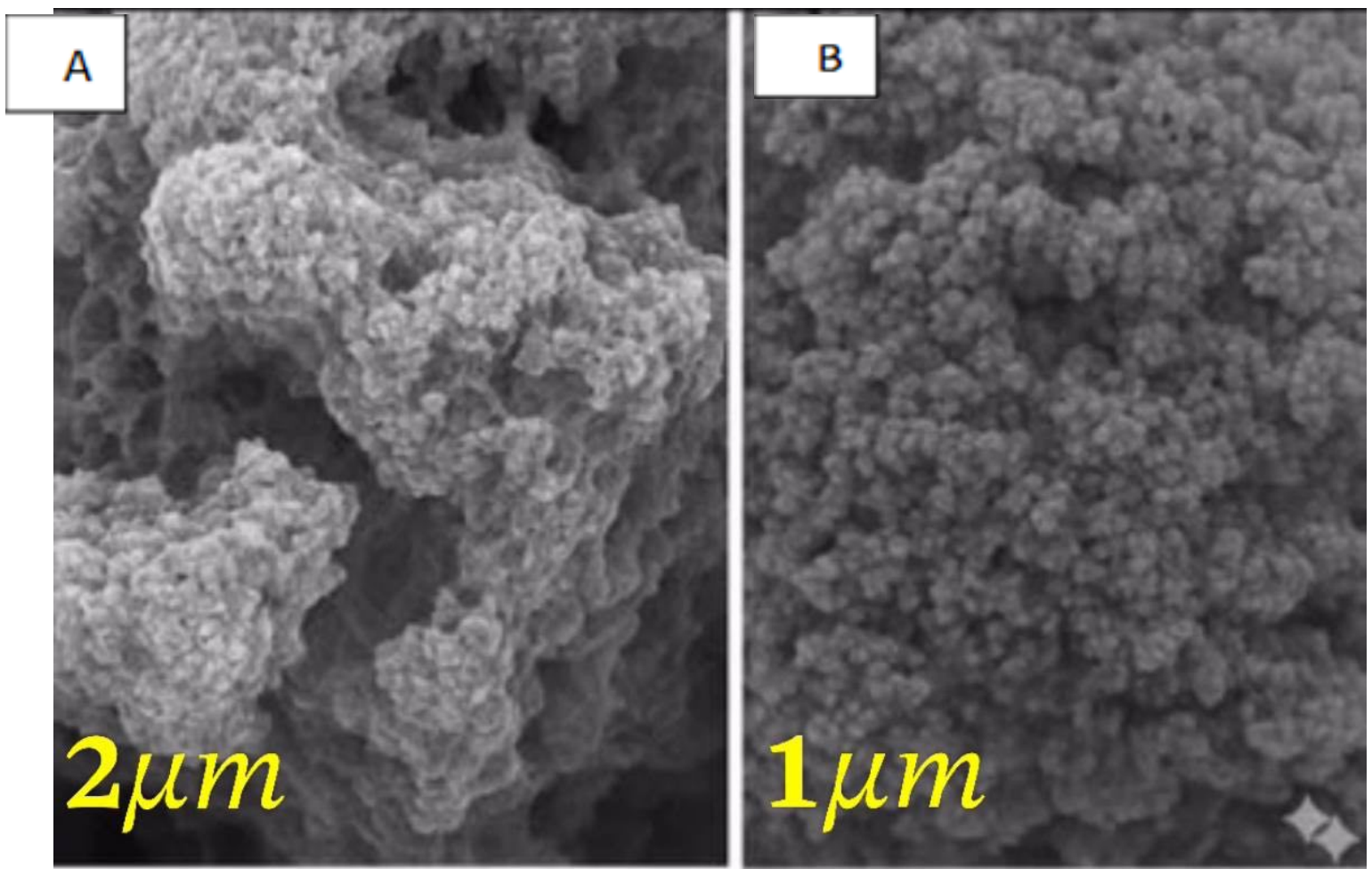

Figure S1 SEM Analysis of curcumin nanoparticles at and 2 μm (A) Scales, 1 μm (B).

2. AFM

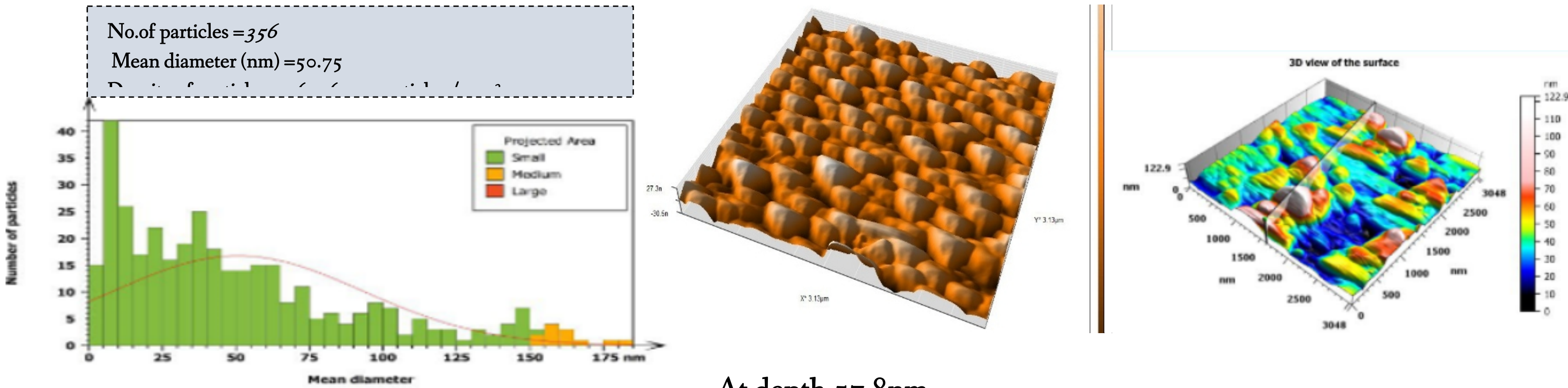

At depth 57.8nm

Figure S2 AFM Analysis of curcumin nanoparticles.

3.2 UV-Vis Spectroscopy Investigation of Molecular Interactions Between Arginine and Nanocurcumin

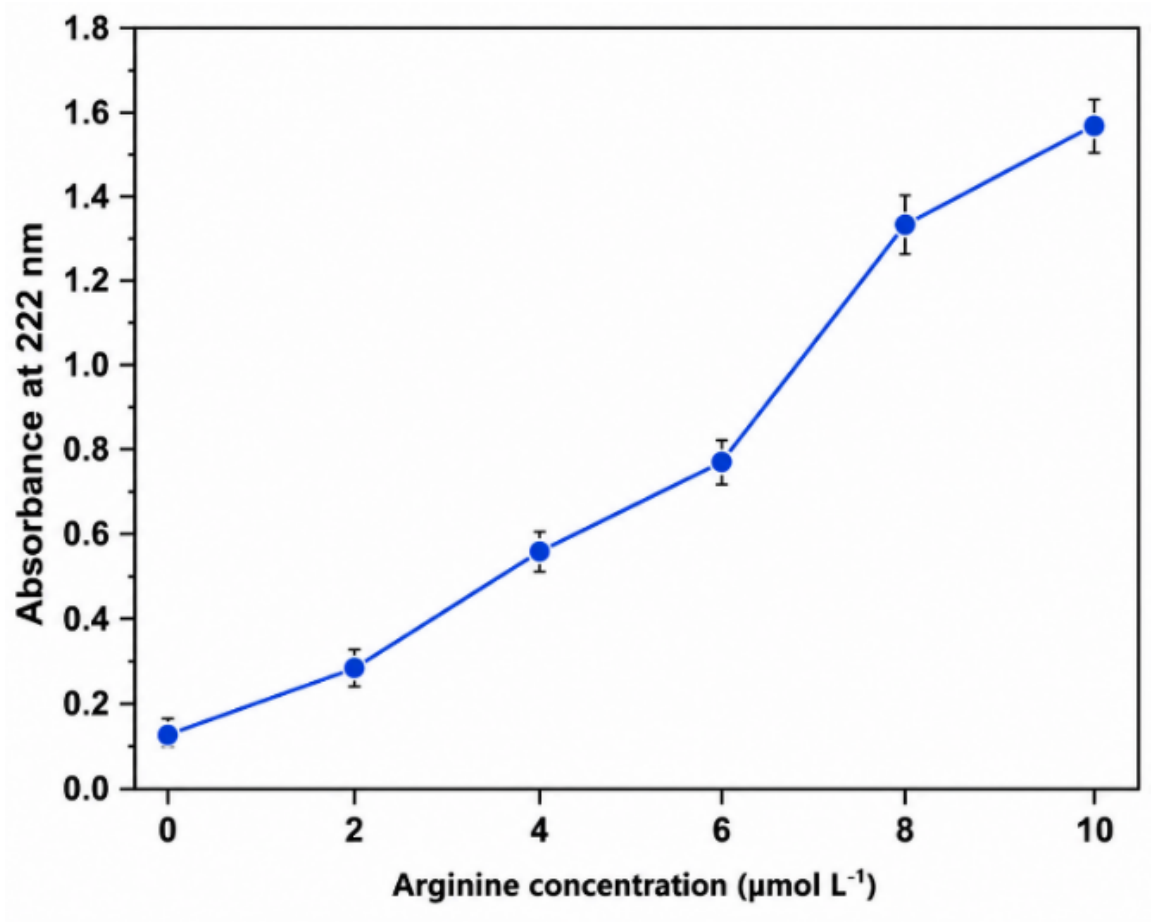

Figure S3. Illustration of UV-Vis spectrophotometric measurements of L-Arginine at λ=222 nm

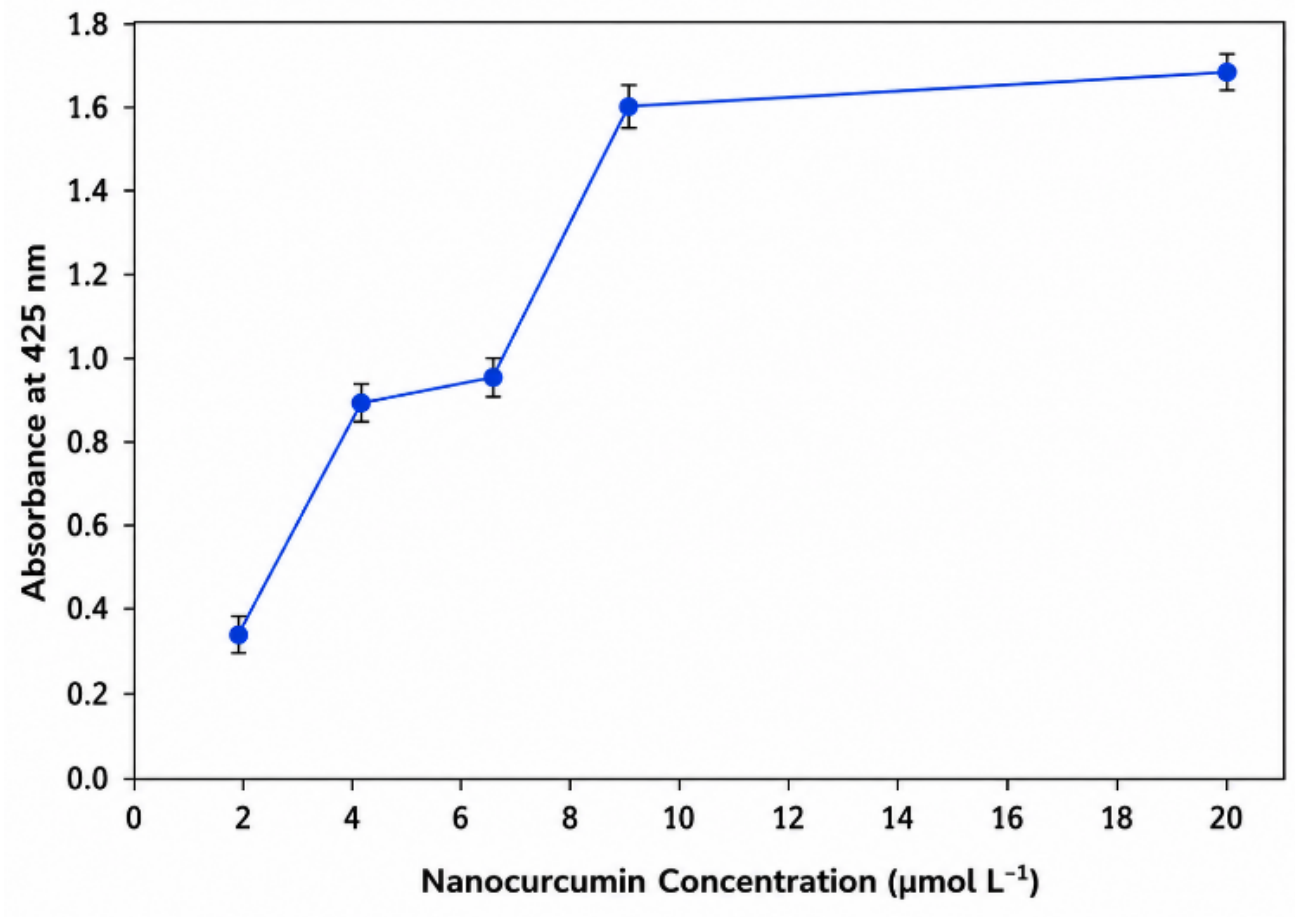

Figure S4. UV-Vis spectrophotometry of Nanocurcumin at λ=425 nm

3.4 Investigation of Nanocurcumin-Arginine compound stability

3.4.1 Effect of Nanocurcumin concentration

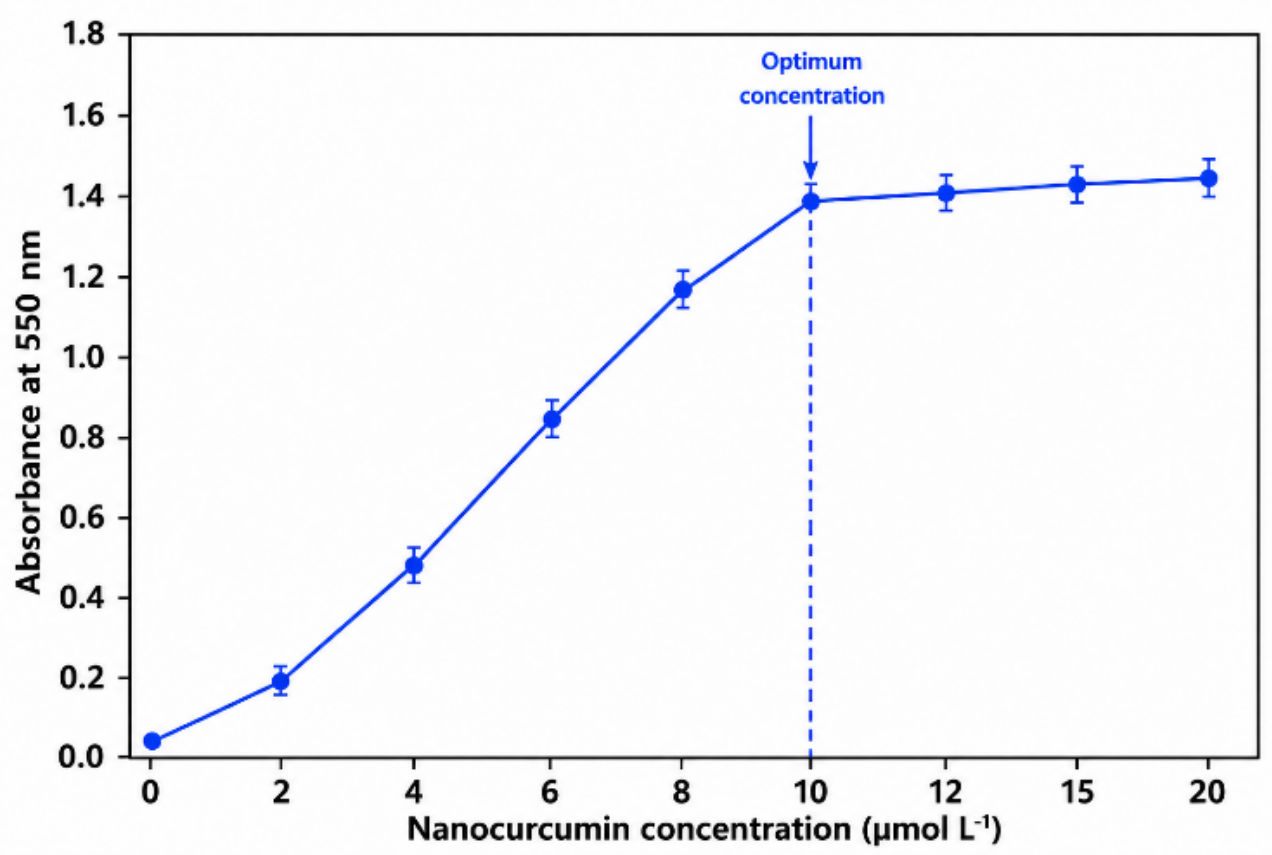

Figure S5. UV-Vis spectrophotometry of Nanocurcumin-Arginine compound at λ=550 nm

3.4.2 Effect of Temperature

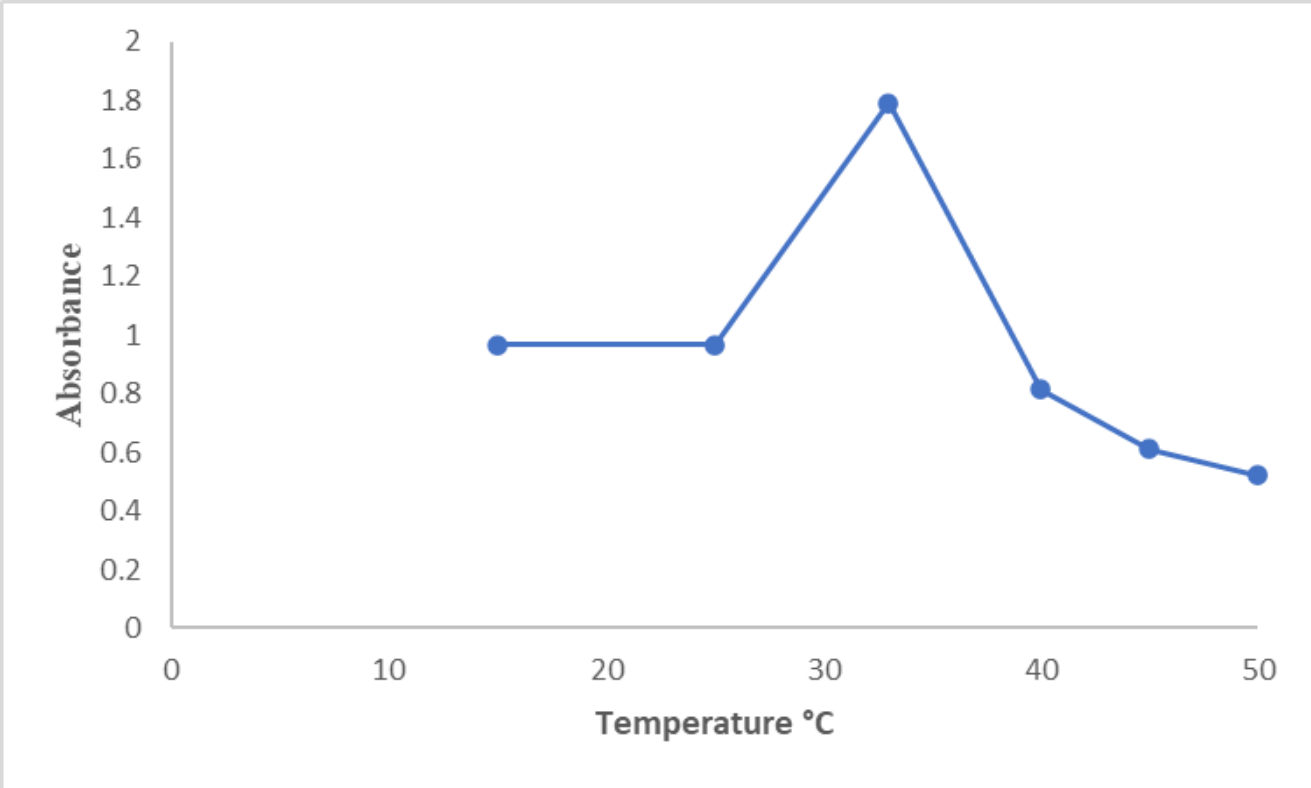

Figure S6. Effect of Temperature on the absorbance of the nano-curcumin–arginine compound

3.4.3 Effect of Time, & UV -Irradiation on Compound Stability

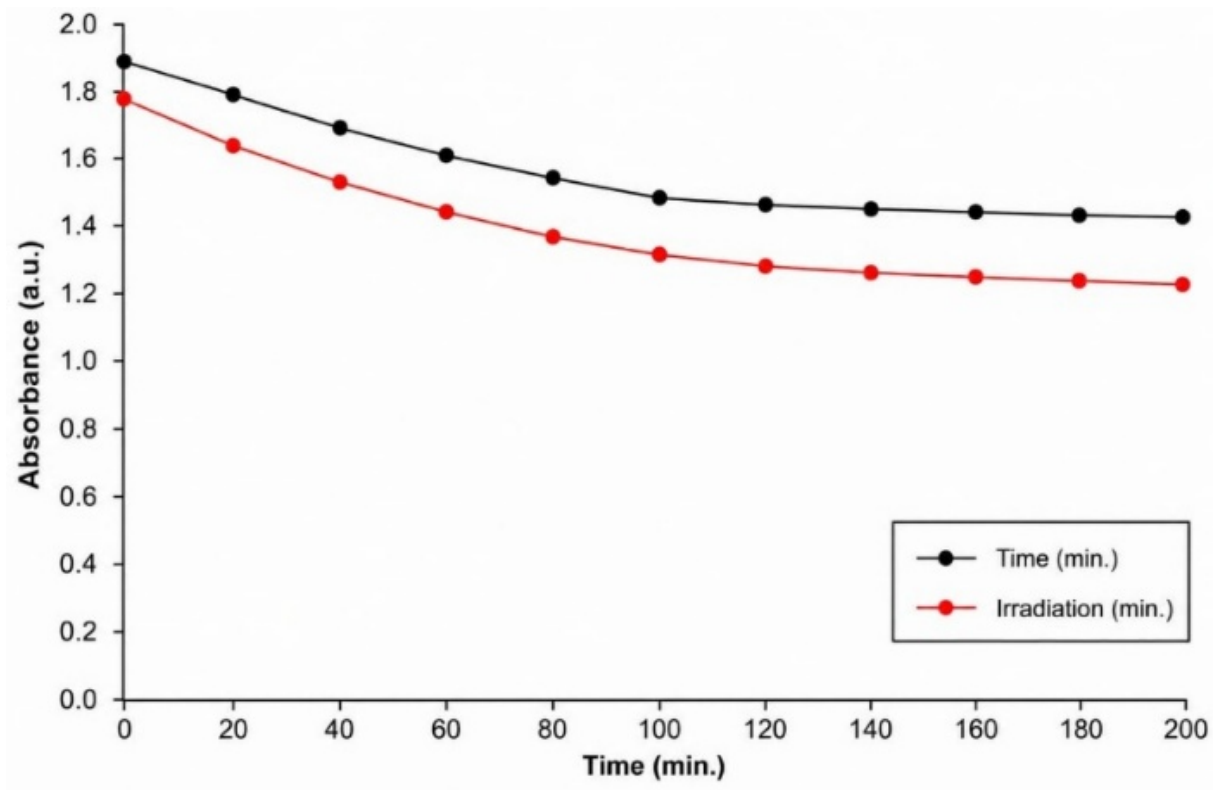

Figure S7. Effect of Time, & irradiation on the stability of the nanocurcumin–L-Arginine compound

3.4.4 Evaluation of Linearity, Precision, and Detection Capability

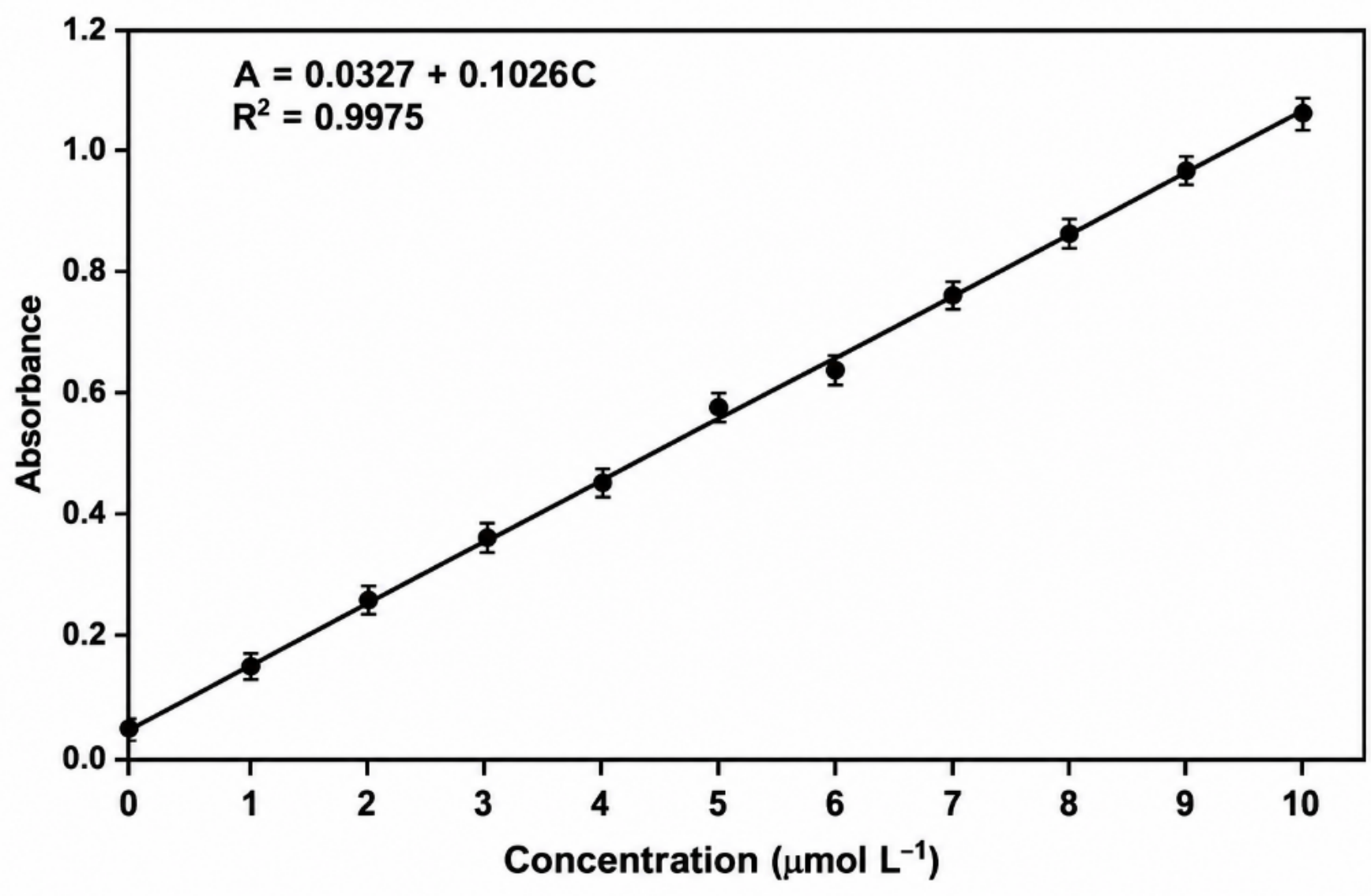

Figure S8 Calibration curve of L-arginine based on the nanocurcumin method (0–10 μM) at 550 nm

Table S1 Analytical performance of nano curcumin- L-arginine compound (λ = 550 nm)

Parameter	Nanocurcumin– L-arginine compound	Unit	Description
Detection wavelength	550	nm	Laser excitation wavelength
Linear range (LDR)	0- 10	μmol L^{-1}	Concentration range with linear response
Calibration equation	A = 0.1026C + 0.0327	-	x: concentration (μmol L^{-1}), y: signal (a.u.)
Sensitivity (m)	0.1026	a.u.· μmol L^{-1}	Slope of calibration curve

Intercept (b)	0.0327	a.u.	Calibration intercept
Correlation coefficient (R^2)	0.9975	—	Linearity of the sensor
Standard deviation of blank(σ)	0.0001	a.u	Noise level (n = 10)
Limit of Detection (LOD)	2.92	nmol L^{-1}	LOD = 3.3σ / m
Limit of Quantification (LOQ)	9.75	nmol L^{-1}	LOQ = 10σ / m
Repeatability (RSD%)	0.2	%	n = 3 at 6 μmol L^{-1}

3.4.5 Analytical Application in Real Samples

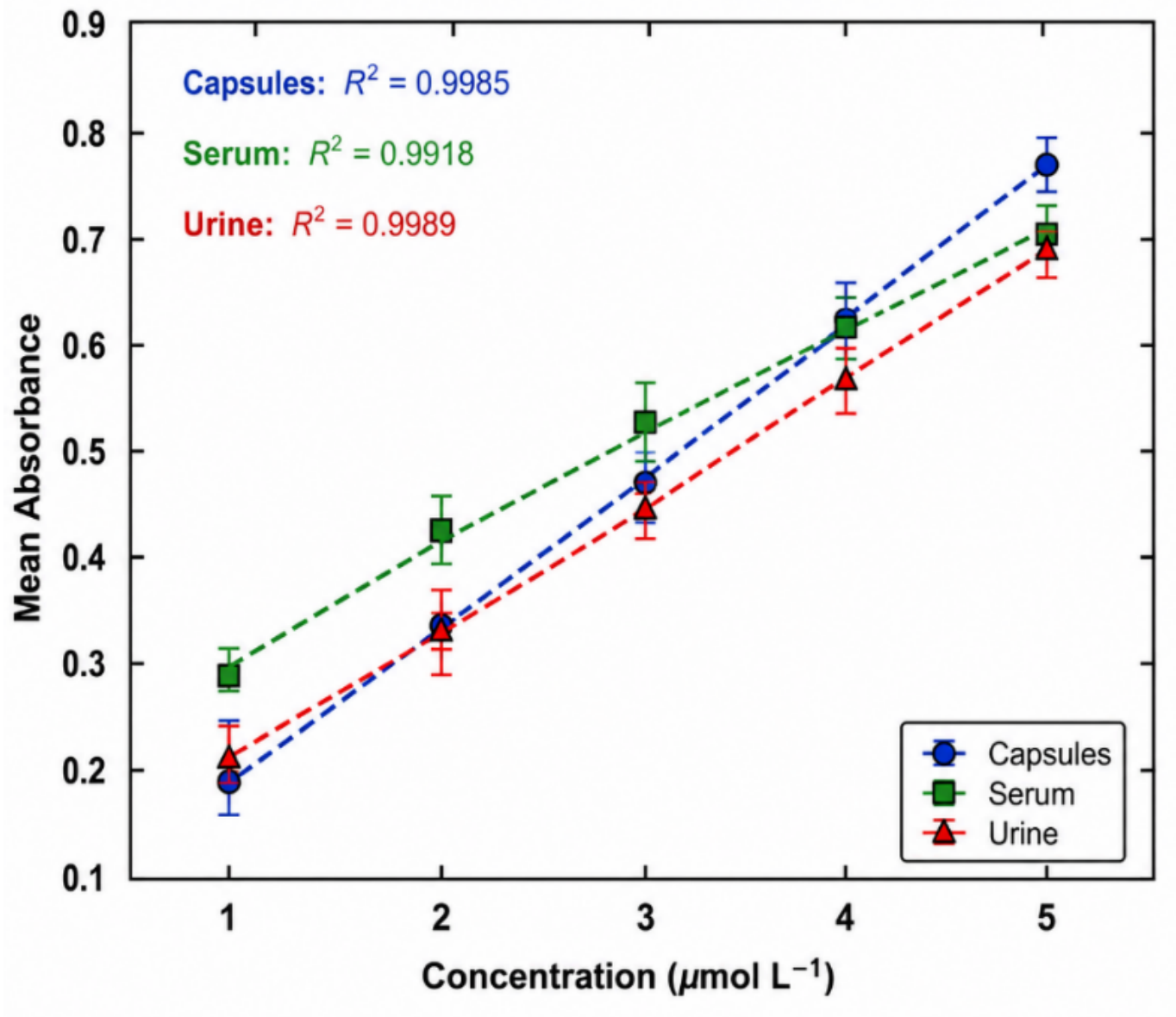

Figure S9. Application of the proposed method for L-arginine determination in capsule, serum, and urine samples at 550 nm.

3.5 Effect of pH on Curcumin Behavior

The pH-dependent behavior of curcumin was investigated over the range **2.16–8.1 (Figure S10).** Noticeable color changes were observed with increasing pH, reflecting the sensitivity of curcumin to protonation–deprotonation equilibria and tautomeric transformations. These changes confirm the potential of curcumin as a pH-sensitive indicator, which may influence its interaction behavior in complex systems.

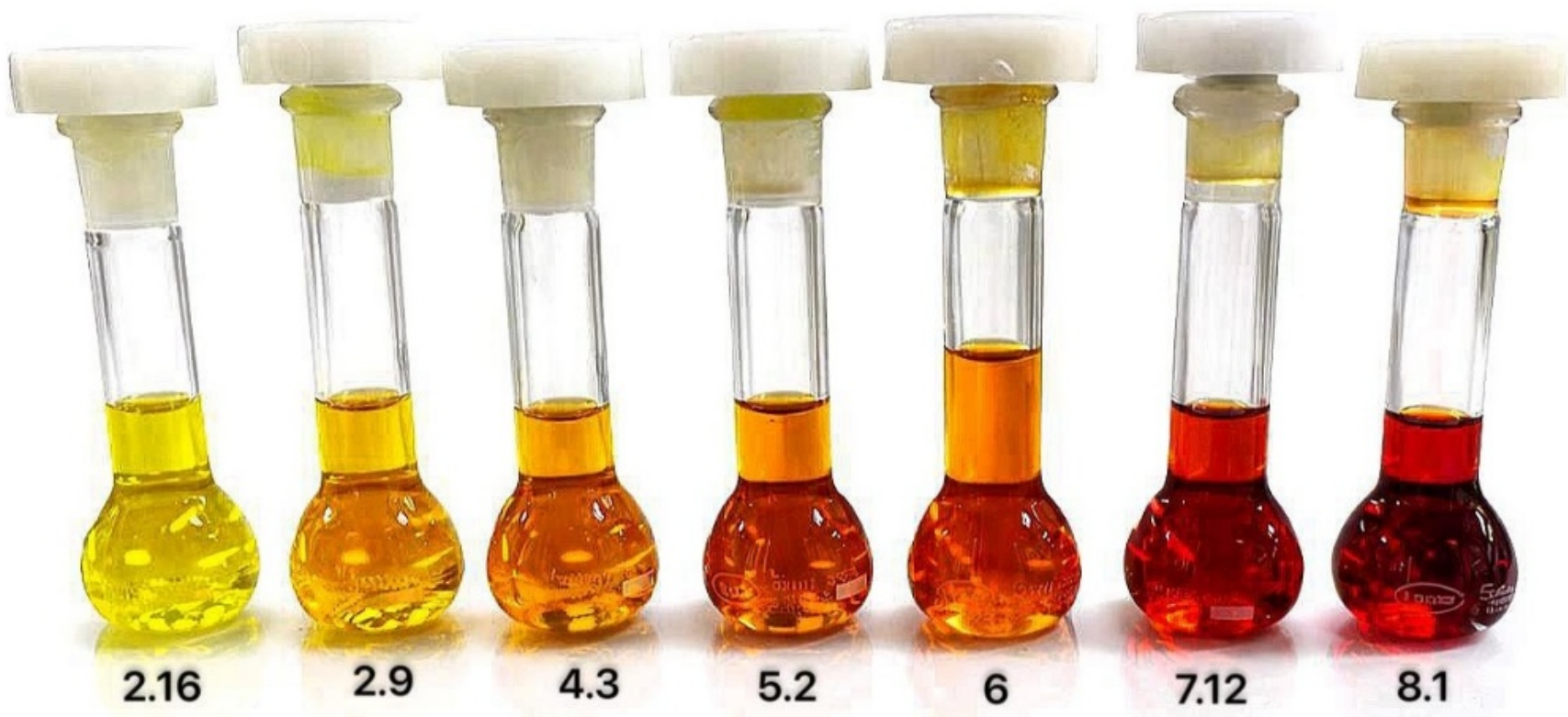

Figure S10. Effect of pH on the nano curcumin only

5. Comprehensive Greenness and Whiteness Assessment of the Microfluidic Platform

Table S2. Input parameters used for GAPI, AGREE, NACaWa, Footprint, and WAC greenness and whiteness software tools.

Assessment Tool	Parameter Category	Specific Input Value / Operational Condition	Eco / Analytical Status
GAPI	Sample Collection & Prep	Direct dilution in water, no chemical extraction	Green (Minimal step)
	Solvents & Reagents	Distilled water (100%), aqueous Nanocurcumin	Green (non-hazardous)
	Instrumentation & Energy	Microfluidic platform / UV-Vis, < 0.1 kWh	Green (Low energy)
	Waste Volume & Treatment	< 1.0 mL total waste per assay, non-toxic	Green (Minimal step)
AGREE	Miniaturization & Throughput	Microchannel flow, high sample throughput	High score contribution
	Reagent Consumption	Microliter volumes (< 50 μL)	Score = 0.88
WAC (RGB)	Green (Environmental)	Reagent toxicity, water solvent, waste volume	Sub-score = 0.90
	Red (Analytical)	Linearity (R^2 > 0.998), LOD, Recovery (95--102%)	Sub-score = 0.90
	Blue (Practicality)	Cost per test (< 0.10), simple operation, speed	Sub-score = 0.90
	Overall Whiteness	Combined RGB Integration Score	Whiteness = 0.88
